# A study on the removal of propyl, butyl, and benzyl parabens *via* newly synthesised ionic liquid loaded magnetically confined polymeric mesoporous adsorbent†

**DOI:** 10.1039/c8ra03408g

**Published:** 2018-07-18

**Authors:** Masrudin Md Yusoff, Noorfatimah Yahaya, Noorashikin Md Saleh, Muggundha Raoov

**Affiliations:** Integrative Medicine Cluster, Advanced Medical and Dental Institute, Universiti Sains Malaysia Bertam 13200 Kepala Batas Penang Malaysia; Research Centre For Sustainable Process Technology, Chemical Engineering Programme, Faculty of Engineering and Built Environment, Universiti Kebangsaan Malaysia 43600 UKM Bangi Selangor Malaysia; University of Malaya Centre for Ionic Liquids, Department of Chemistry, Faculty of Science, Universiti Malaya Kuala Lumpur 50603 Malaysia muggundha@um.edu.my; Department of Chemistry, Faculty of Science, Universiti Malaya Kuala Lumpur 50603 Malaysia

## Abstract

This study investigated the effectiveness of ionic liquids (ILs) loaded onto the surface of a polymeric adsorbent (βCD-TDI) grafted with modified magnetic nanoparticles (MNPs) *via* an analysis of water treatment, which resulted in high removal of selected endocrine-disrupting chemicals (parabens). The syntheses of MNPs, MNP-βCD-TDI, and IL-MNP-βCD-TDI were characterised and compared using Fourier transform infrared (FT-IR) spectroscopy, carbon–hydrogen–nitrogen (CHN) analysis, vibrating sample magnetometry (VSM), scanning electron microscopy (SEM), transmission electron microscopy (TEM), the Brunauer–Emmett–Teller (BET) method, thermogravimetric analysis (TGA), and X-ray diffraction (XRD). The results of SEM and TEM indicated that the pore size distribution exhibited mesoporous characteristics with a small surface area (BET analysis: 42.95 m^2^ g^−1^). Furthermore, a preliminary sorption experiment demonstrated the ability of IL-MNP-βCD-TDI to enhance not only the sorption capacity, but also the removal of propyl paraben (PP), butyl paraben (BP), and benzyl paraben (ArP). The adsorption process appeared to be pH-dependent, and hence the optimum pH of 6 was selected for a subsequent batch adsorption study of all the studied parabens with an equilibrium time of 80 min. Next, in an attempt to investigate the interactions that occur between the adsorbent and the adsorbates, adsorption kinetics and isotherm studies were performed. All the studied parabens were found to best fit pseudo-second-order kinetics and the Freundlich isotherm with *R*^2^ > 0.98 at room temperature (298 K). The interaction of the host–guest inclusion complex and the π–π interaction between βCD and a selected paraben compound (ArP) were identified by performing ^1^H nuclear magnetic resonance (NMR), together with ultraviolet-visible (UV-vis) spectroscopic analysis. Finally, the adsorption efficiency of the developed material was practically tested on tap water, drain water, and industrial wastewater, which revealed a significant removal of parabens of up to 60–90% in comparison with a prior analysis.

## Abstract 2

The main interaction between the synthesized material and the targeted paraben compounds.

## Graphical abstract

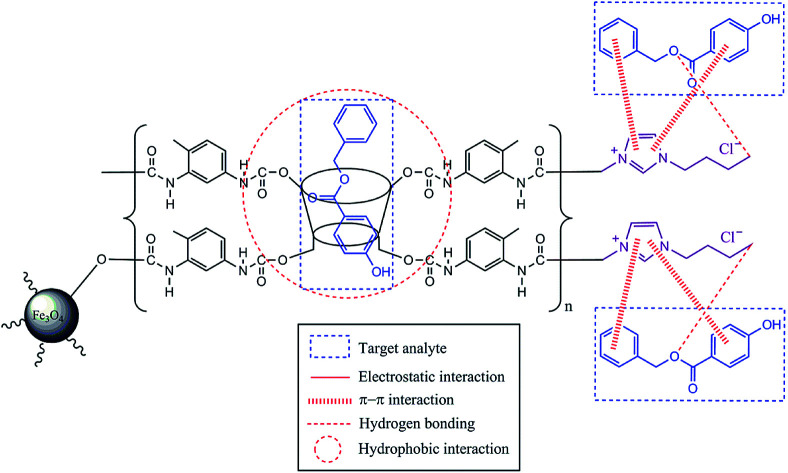

## 1. Introduction

Magnetic nanoparticles (MNPs) have appeared to have great potential as sorbents in a number of applications studied by researchers. Besides, they have the ability to amalgamate easily with any pollutant in huge amounts.^1^ In fact, the term MNPs refers to a class of nanoparticle agglomerates, which resemble particles that are small in size and can be manipulated using an external magnetic field.^2^ In addition, MNPs have been widely employed by researchers owing to their nanoscale sizes, which are typically around 10–20 nm and correspond to those of mesoporous types of materials. Nanomaterials or nanoparticles are materials with two or more dimensions and unique size dependence in terms of physical and chemical properties.^3^ Therefore, in order to develop a new promising adsorbent, MNPs alone are insufficient to maximize the adsorption capacity for targeted analytes and require several modifications of the surface of MNPs with other materials.^4–12^ This may enable exceptional outcomes, especially when dealing with small sample volumes, owing to the large surface areas.^13^

Furthermore, the growing interest in the field of supramolecular chemistry has allowed this study to be carried out. As such, this study represents an experimental study in which MNPs were coated with cyclodextrin (CD) as an effective adsorbent for the removal of parabens. CDs, which are also known as cycloamyloses, are a family of compounds that are composed of sugars bound together in a ring (cyclic oligosaccharides). The widely used applications of CDs in pharmaceuticals, food, drug delivery, and chemicals are highly desirable owing to the unique characteristics of their structure. CDs are produced by cyclomaltodextrin glucanotransferase, whereby three common cyclodextrins are available with 6, 7 or 8 d-glucopyranosyl residues, namely, α-CD (6 glucose units), β-CD (7 glucose units), and γ-CD (8 glucose units), respectively, which are linked in a ring by α-1,4-glycosidic linkages.^14^ As such, β-cyclodextrin (βCD) is a natural starch derived from molecules that is a torus-shaped cyclic oligosaccharide with an internal hydrophobic cavity.^15^

βCD was selected for the experimental work in this study because it is low in cost and has the ability to form solid inclusion complexes^16,17^*via* various kinds of interactions^18^ with different types of guest compounds. Furthermore, βCD can alter its properties *via* polymerization upon cross-linking, which refers to the process of chemically joining two or more molecules by a covalent bond and transforming the molecule into a water-insoluble molecule.^19^ βCD can also be cross-linked by a reaction between hydroxyl groups on the chain and a coupling agent to form a water-insoluble network.^20,21^ Owing to the properties of CD, which contains many hydroxyl groups, the best cross-linking agent is a diisocyanate linker such as toluene 2,4-diisocyanate (TDI)^22–26^ because of its reactivity towards hydroxyl groups. Hence, TDI was selected to be used in this study in order to transform the βCD molecule into a three-dimensional network polymer (βCD-TDI).

Furthermore, CD polymers have also emerged as an area of interest among academicians, especially in combination with MNPs^27–35^ such as those with the formula Fe_3_O_4_.^36^ MNP surface modification appears to be a frequently used technique for retaining their internal superior magnetic properties upon combination with polymeric adsorbents. Therefore, the aggregation of MNPs and the transformation of magnetite (Fe_3_O_4_) into maghemite (γ-Fe_2_O_3_) are attributable to the reaction of Fe(ii) cations with oxygen, which can be prevented.^29^ The unique characteristics of combinations of these types of adsorbents are the inner MNP itself, which possesses the ability to sense and respond to an external magnetic field, whereas the outer CD polymer functions as an inclusion site and a specific container for the adsorption of targeted analytes. Thus, the modification of MNPs with a βCD polymer seems to provide an effective adsorbent for the removal of organic compounds.

In addition, in order to increase the selectivity for targeted analytes, the introduction of an ionic liquid (IL) onto a polymer surface has sparked interest in a number of studies. In conjunction with the properties of βCD, a number of methods have been developed that promote the incorporation of βCD with an IL, mainly because the presence of an IL has been proven to enable more chemical interactions between targeted analytes.^37–42^ The term IL refers to a type of salt in the form of a liquid below 100 °C or even at room or ambient temperature, of which the latter are also known as room-temperature ionic liquids (RTILs).^43^ RTILs have begun to gain wide recognition as novel solvents within the field of chemistry owing to the unique non-volatility, non-flammability, low viscosity, and electrochemical stability of ILs, which are beneficial in various types of applications, especially in supramolecular materials.^44,45^ Therefore, a new approach has been developed in this study by loading an IL onto the surface of MNP-βCD-TDI so as to create a new magnetically confined polymeric mesoporous adsorbent loaded with an ionic liquid (IL-MNP-βCD-TDI).

The newly developed material can be used to remove endocrine-disrupting chemicals (propyl, butyl, and benzyl parabens) from the environment, especially emerging pollutants^46^ that may cause serious diseases such as cancer.^47^ Contamination of environmental bodies of water has always been associated with uncontrolled disposal of wastewater from industries, which has an adverse impact upon the health of organisms.^48–50^ Therefore, adsorption techniques that integrate such a novel approach can be evaluated *via* an adsorption process. This study has been divided into three principal parts, namely, (1) synthesis of adsorbents, (2) characterisation of adsorbents, and (3) batch removal experiments. The adsorption process was optimised in order to ensure exceptional adsorption of the targeted analytes, as well as to study the mechanism of the interaction between the adsorbent and the adsorbates. Finally, the applicability of the adsorbent was confirmed by treating real environmental water samples.

## 2. Experimental

### 2.1 Materials and chemicals

Standard samples of three paraben compounds, namely, propyl paraben (PP), butyl paraben (BP), and benzyl paraben (ArP), 1-butyl-3-methylimidazolium chloride (BMIM-Cl), toluene-2,4-diisocyanate (TDI), and anhydrous dimethyl sulfoxide-d_6_ (DMSO) were purchased from Sigma-Aldrich (St. Louis, MO, USA). Iron(ii) chloride tetrahydrate (FeCl_2_·4H_2_O) and iron(iii) chloride hexahydrate (FeCl_3_·6H_2_O) were purchased from R&M Chemicals (Essex, UK), whereas β-cyclodextrin (βCD, 99%) was commercially available and was purchased from Acros (Hungary). Moreover, acetonitrile (ACN), methanol (MeOH) (HPLC grade, 99.7%), acetone (technical grade), and ammonia solution (25%) were supplied by Friendemann Schmidt (Parkwood, Australia), whereas anhydrous *N*,*N*-dimethylformamide (DMF) was purchased from Merck (Kenilworth, NJ, USA). Analytical-grade absolute ethanol (denatured, 99.7%) was purchased from J. Kollin Chemicals (Midlothian, UK), whereas deionised (DI) water (18.2 MΩ cm) was provided by a Sartorius Milli-Q system (Aubagne, France).

### 2.2 Part 1: Synthesis of adsorbents

The adsorbents were prepared in accordance with a procedure detailed in a prior work.^51^ Co-precipitation was performed under a non-oxidising nitrogen atmosphere in order to generate bare MNPs with a molar ratio of 1 : 2. Next, FeCl_2_·4H_2_O (0.86 g) and FeCl_3_·6H_2_O (2.34 g) were dissolved in 40 mL of deionised water and stirred for 30 min at 1200 rpm. The solution temperature was increased to 90 °C and 5 mL of NH_4_OH (25%) was added directly, which was then stirred for an hour. In order to remove any unreacted chemicals, the nanoparticles that formed were washed with deionised water five or six times. The resulting product was isolated using an external magnet and dried in a vacuum oven at 40 °C.

Next, a CD polymer was synthesised in accordance with a literature method.^37,39^ βCD (2 g, 1.76 mmol) was dissolved in 50 mL of anhydrous DMF at room temperature. In an inert atmosphere, the TDI linker (2.54 mL, 17.6 mmol) was added and the reaction mixture was stirred for 24 h at 70 °C. The addition of excess acetone caused the precipitation of βCD-TDI. In order to remove residual DMF from the polymer, the precipitate that formed was stirred and allowed to settle in acetone for 10 min. Then, the reaction mixture was filtered and washed with acetone and deionised water several times to remove unreacted cross-linker, and was later dried overnight in a vacuum.

After the drying process, MNP-βCD-TDI was synthesised *via* a one-step co-precipitation method^34,52^ by dissolving FeCl_2_·4H_2_O (0.86 g, 4.33 mmol), FeCl_3_·6H_2_O (2.34 g, 8.66 mmol), and βCD-TDI (1.5 g, 1.30 mmol) in 40 mL of deionised water in a molar ratio of 1 : 2 : 0.3, and the reaction mixture was vigorously stirred at a speed of 1200 rpm at 90 °C for 30 min. Next, in order to generate conglomerated nanoparticles, 5 mL of NH_4_OH (25%) was added after the solution was heated to 90 °C, and the reaction mixture was continuously stirred for 1 h at 90 °C in an inert atmosphere. In order to remove any unreacted chemicals, the nanoparticles that formed were washed with deionised water five or six times. The resulting product was isolated using an external magnet and was later dried in a vacuum oven at 40 °C.

**Scheme 1:**
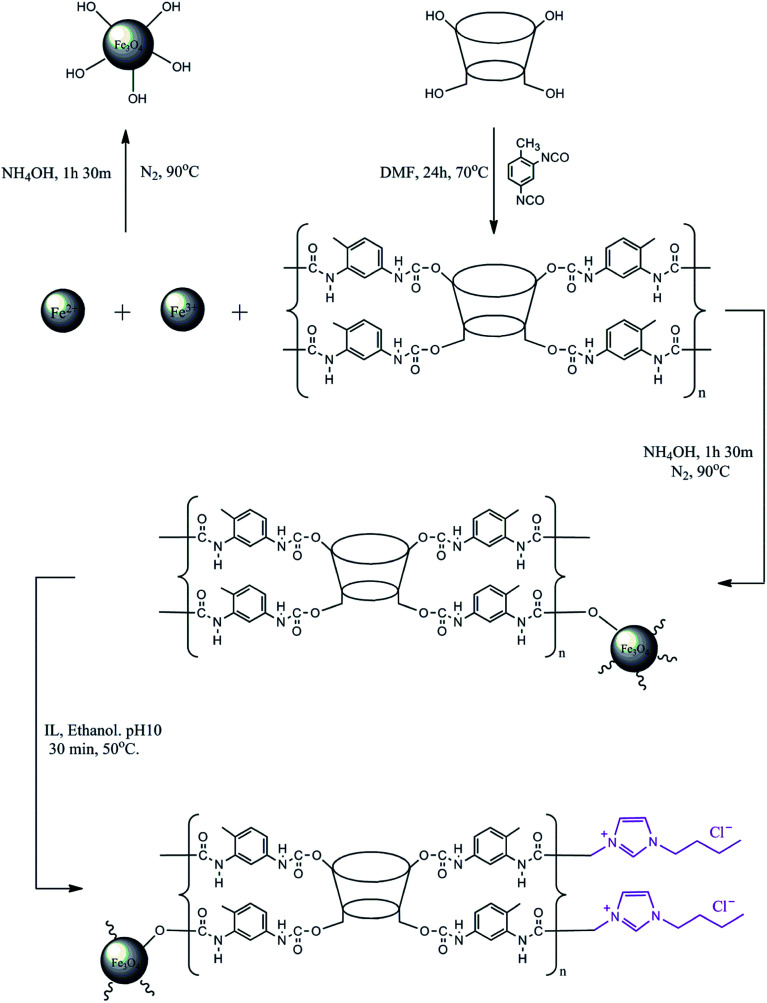
Complete illustration of the synthesised materials.

The modification of the surface of MNP-βCD-TDI using an IL was adopted from prior works.^32,53,54^ BMIM-Cl (4.0 g L^−1^) was diluted using 25 mL of absolute ethanol (99.7%) with the addition of NaOH (0.01 mol L^−1^) while the pH of the solution was adjusted to 10.0. After that, MNP-βCD-TDI (0.5 g) was added to the solution and was stirred at 50 °C for 30 min at 280 rpm to allow all the nanoparticles to be suspended in the solution and adhere to the surface of MNP-βCD-TDI in the presence of the IL. The nanoparticles that formed were then washed with 50 mL of ACN and deionised water three times. Finally, the resulting product was isolated using an external magnet and was later dried in a vacuum oven at 40 °C. A complete illustration of the synthesised materials is given in Scheme 1.

### 2.3 Part 2: Characterisation of adsorbents

Fourier transform infrared spectroscopy (FT-IR; Thermo Nicolet, Madison, WI, USA) was carried out in the wavenumber range between 4000 cm^−1^ and 400 cm^−1^. Moreover, carbon–hydrogen–nitrogen analysis (PerkinElmer 2400 Series II CHN analyser, Massachusetts, USA) was performed to determine the amounts of C, H, and N present. Next, the magnetisations of the bare and both modified MNPs were measured using a vibrating sample magnetometer (Lake Shore 7404 series, McCorkle Boulevard, Westerville, OH, USA). The morphology and particle size, on the other hand, were determined using a scanning electron microscope (Quanta FEG650, Oxford Instruments, Hillsboro, USA) and a transmission electron microscope (FEI CM12, Hillsboro, USA). After that, a surface area analyser was employed to determine the surface area and porous properties of the materials *via* nitrogen adsorption–desorption analysis at 77 K using the Brunauer–Emmett–Teller (BET) method (Quantachrome, Boynton Beach, FL, USA). The thermal stability of the synthesised materials was later investigated by performing thermogravimetric analysis (TGA; PerkinElmer TGA-STA 1500, Massachusetts, USA) at a heating rate of 10 °C min^−1^ between 30 and 900 °C under a nitrogen atmosphere. X-ray diffraction patterns were recorded using an Empyrean X-ray diffractometer (PANalytical, Almelo, Netherlands) from 2*θ* = 10° to 90° with Cu Kα radiation (*λ* = 1.5418 Å) at a scan rate of 0.02° s^−1^. Finally, nuclear magnetic resonance (NMR, Bruker Avance III 700 MHz, Rheinstetten, Ettlingen, Germany) was employed to characterise the formation of the inclusion complex of βCD with ArP.

### 2.4 Part 3: Batch removal experiments

#### 2.4.1 Preliminary batch study

The prepared adsorbents were examined in adsorption studies in order to compare the performance of IL-MNP-βCD-TDI with that of MNP-βCD-TDI and native MNPs. The removal conditions were: temperature, 25 °C; analyte solution, 10 mL; sorbent dosage, 20 mg; equilibrium time, 2 h at 250 rpm.

#### 2.4.2 Sorption experiments

PP, BP, and ArP were selected as analytes for batch adsorption experiments, and several parameters, such as the effect of pH, kinetics, effect of contact time, effect of concentration, sorbent dosage, and reusability, were optimised by employing a batch method using 20 mg of IL-MNP-βCD-TDI, 10 mL of an aqueous solution that contained the analytes of interest at a known concentration (10 mg L^−1^) in a tightly sealed vial, and a shaking time of 2 h (180 rpm) at room temperature. After the adsorption process, the adsorbent was separated using an external magnetic field and was filtered prior to analysis with an ultraviolet-visible spectrometer (PerkinElmer Lambda 25, Massachusetts, USA) equipped with 10 mm quartz cuvettes (PerkinElmer, Massachusetts, USA) at wavelengths of 256 nm (PP and BP) and 257 nm (ArP). All the samples were tested in triplicate, whereas the removal efficiency (*R*%) was calculated using the following equation:1
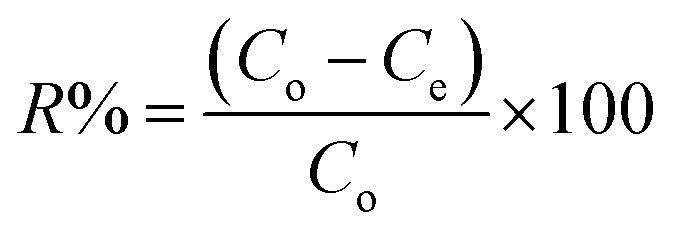


The amount of the analytes adsorbed per unit mass of the adsorbent (*q*_e_) was calculated as follows:2
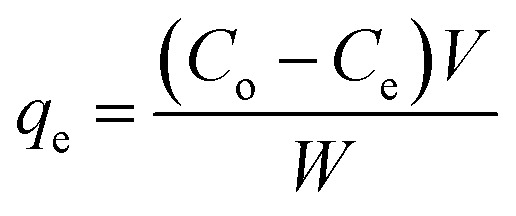
where *C*_o_ and *C*_e_ are the initial and equilibrium concentrations of the solution (mg L^−1^), respectively, *V* (L) denotes the volume of the solution, whereas *W* (g) refers to the mass of the dry adsorbent used.

#### 2.4.3 Effect of solution pH

Adsorption was performed at various pH values that ranged from 3 to 10 at room temperature. The desired pH was adjusted with 0.01 M HCl and 0.01 M NaOH using a pH meter (OHAUS Starter 3100, Ohio, USA). The initial concentrations of the analytes were fixed at 10 mg L^−1^ in 10 mL of an aqueous solution of the analytes with a sorbent dosage of 20 mg, which was agitated at 250 rpm for 120 min.

#### 2.4.4 Effect of contact time

The effect of the contact time on the removal of parabens using IL-MNP-βCD-TDI was investigated at various time intervals of between 0 and 120 min at room temperature. The initial concentrations of the analytes were fixed at 10 mg L^−1^ in 10 mL of an aqueous solution of the analytes with a sorbent dosage of 20 mg at a pH of 6 for all the studied analytes, and the solution was agitated at 250 rpm.

#### 2.4.5 Effect of initial concentration

Equilibrium studies were performed at various initial concentrations of the analytes within the range of 0–100 mg L^−1^ in 10 mL of an aqueous solution of the analytes with a sorbent dosage of 20 mg at a pH of 6 at three different temperatures, namely, 298 K, 318 K, and 338 K, and the solution was later agitated at 250 rpm for 80 min.

#### 2.4.6 Effect of sorbent dosage

The effect of the sorbent (IL-MNP-βCD-TDI) dosage on the adsorption process was determined by varying the sorbent dosage from 20 to 120 mg with an analyte concentration of 80 mg L^−1^ at a pH of 6 and a temperature of 298 K. The appropriate sorbent dosage of IL-MNP-βCD-TDI was weighed under a tightly sealed vial, and the solution was agitated at 250 rpm for 80 min. The adsorption capacity (mg g^−1^) per unit mass was calculated using eqn (2).

#### 2.4.7 Preparation of real samples

Three types of water samples, namely, tap water, drain water, and industrial wastewater, were collected and filtered using 0.45 μm membrane filters and were then stored in the dark at 4 °C. After that, the samples were spiked at a concentration of 80 mg L^−1^ in 10 mL and were directly added to a vial that contained 100 mg of the sorbent for a further adsorption process. Batch experiments were conducted in triplicate (*n* = 3) so as to ensure the accuracy and precision of the results.

#### 2.4.8 Regeneration of IL-MNP-βCD-TDI adsorbent

The reusability of IL-MNP-βCD-TDI was determined five times *via* adsorption–desorption cycles. Firstly, after the adsorption process, the wet sorbent was collected and immersed in 5 mL of deionised water, which was subsequently vortexed for 1 min, and the sorbent was isolated using an external magnet. A similar procedure was repeated by replacing the deionised water with ACN, and the sorbent was then dried for 1 h in a vacuum oven.

#### 2.4.9 Synthesis and characterization of βCD–ArP inclusion complex

The inclusion complex was prepared *via* a conventional kneading method.^55^ Equimolar amounts of βCD and ArP were kneaded for approximately 30 min with a mortar and pestle in a minimal amount of analytical-grade absolute ethanol (denatured, 99.7%), which was purchased from J. Kollin Chemicals (Midlothian, UK), to produce a homogeneous paste and dried to a constant mass. After drying, a white powdery complex was obtained and characterized using ^1^H NMR. The calculated yield was approximately 65.8%.

#### 2.4.10 Preparation of βCD-ArP for spectroscopic studies

A 2 mL portion of 0.01 mM ArP, 3.2 mL of a 0.004 M solution of βCD, and 2 mL of a buffer solution (pH 7) were transferred accurately to a 10 mL volumetric flask and diluted to the mark using deionised water, which was then mixed well and agitated for 5 min *via* ultrasound. After that, the solution was left for 30 min at room temperature. The absorption spectrum of the βCD–ArP complex was recorded against a blank reagent, which was prepared with the same reagent concentration but in the absence of ArP. Furthermore, the absorption spectra of βCD and ArP were recorded in accordance with the same procedure. All absorbances were measured at 257 nm against the blank reagent. Moreover, to generate a constant curve, the concentration of ArP was kept constant at 0.01 mM but the concentration of βCD was varied (0.004, 0.005, 0.007, 0.009, and 0.01 M). This procedure was repeated so as to obtain three or more absorbance values for each βCD concentration that was studied.

## 3. Results and discussion

### 3.1 Characterisation of materials

**Fig. 1:**
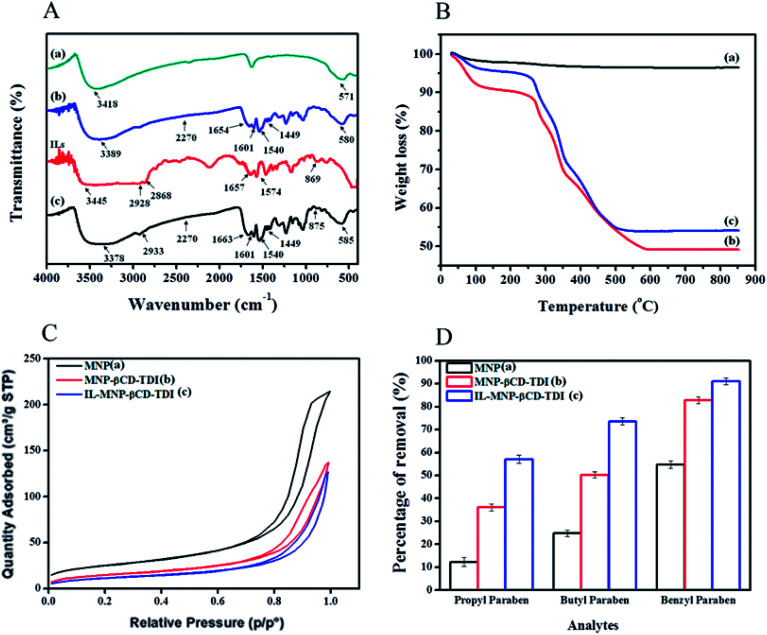
(A–C) Present the results of the characterisation of the synthesised adsorbents, whereas (D) shows the results of preliminary batch adsorption experiments for comparison between (a) MNPs, (b) MNP-βCD-TDI, and (c) IL-MNP-βCD-TDI.

The results of the FT-IR and TGA analyses and the BET hysteresis loops in this study are illustrated in Fig. 1, whereas Table 1 summarises the results of the (a) FT-IR, (b) TGA, (c) BET and (d) CHN analyses in detail. The FT-IR spectra (Fig. 1A) of MNPs, MNP-βCD-TDI, IL (BMIM-Cl), and IL-MNP-βCD-TDI were recorded using the KBr method. As a result, the presence of magnetic properties in the MNPs corresponding to curves (a), (b), and (c) is proven by the appearance of the Fe–O peak, which represents the tetrahedral form of pure MNPs, at about 570–590 cm^−1^. Furthermore, the absence of a peak at 2270 cm^−1^ (corresponding to the isocyanate group) in curves (b) and (c) indicates the success of the polymerisation process, together with the presence of a peak due to carbamate (NHCOO) linkages at 1654 cm^−1^.^56^ In the case of Fe_3_O_4_, the broad absorption peak at around 3400 cm^−1^ signifies the presence of surface hydroxyl groups (O–H stretching), whereas the broad –OH stretching band of MNP-βCD-TDI at about 3300–3400 cm^−1^ is attributable to multiple –OH functional groups and appears to decrease owing to cross-linking with TDI. Moreover, as for the FT-IR spectrum of IL-MNP-βCD-TDI in curve (d), the peak broadening observed at 2900–3400 cm^−1^ indicates the successful loading of the IL onto MNP-βCD-TDI.

**Table 1:** Physicochemical properties determined by FT-IR, TGA, BET and CHN analyses

Characteristic	MNP	MNP-βCD-TDI	IL-MNP-βCD-TDI
**(a) FT-IR spectra (cm** ^**−1**^ **)**			
N–H and O–H stretching	3418	3389	3378 (imidazole)
Fe–O stretching vibration	571	580	585
Absence of N=C=O group	—	2270	2270
NHCO, carbamate linkages	—	1654, 1540	1663, 1540
Aromatic group in TDI	—	1601, 1449	1601, 1449
C=C and C=N stretching	—	—	1663, 1601
C–N stretching vibration	—	869	875

**(b) TGA analysis**			
Water loss/–OH groups	30–160 °C (3%)	—	—
Water loss/–OH groups	—	30–110 °C (8%)	—
Carbamate linkages and βCD	—	250–350 °C (21%)	—
βCD	—	350–600 °C (19%)	—
Water loss/–OH groups	—	—	30–110 °C (8%)
Carbamate linkages and βCD	—	—	250–350 °C (21%)
βCD/combustion of IL	—	—	350–500 °C (19%)

**(c) BET analysis**			
Surface area (m^2^ g^−1^)	90.14 m^2^ g^−1^	54.72 m^2^ g^−1^	42.95 m^2^ g^−1^
Pore volume (cm^3^ g^−1^)	0.33 cm^3^ g^−1^	0.21 cm^3^ g^−1^	0.19 cm^3^ g^−1^
Pore size (nm)	14.72 nm (mesopore)	15.49 nm (mesopore)	18.27 nm (mesopore)
N_2_ adsorption/desorption isotherm	Type IV isotherm with H3-type hysteresis loop	Type IV isotherm with H3-type hysteresis loop	Type IV isotherm with H3-type hysteresis loop

**(d) CHN analysis (%)**			
Carbon (C)	0.23	18.96	47.75
Hydrogen (H)	0.23	1.92	5.28
Nitrogen (N)	0.05	3.79	9.32

VSM and XRD analyses were reported in a prior work^51^ in which the magnetic behaviour of the synthesised adsorbent was determined *via* VSM analysis. In order to separate the MNPs from the solution, a magnetisation (Ms) of 16.3 emu g^−1^ seemed scientifically adequate because a higher Ms value facilitates the separation of MNPs from the solution.^57^ The maximum Ms value of the MNPs in this study was 60.6 emu g^−1^ (a), but in the presence of the polymeric materials coated on the surface of the MNPs the value of Ms decreased to 46.8 emu g^−1^ (b).^4,58^ Upon loading with the IL, the value of Ms appeared to be lower than that of the first coating, namely, 25.0 emu g^−1^ (c). This confirms the successful coating of the IL onto the surface of MNP-βCD-TDI.

The crystal structures of all the synthesised adsorbents were successfully analysed *via* XRD. The characteristic diffraction peaks of the MNPs appeared to correspond to those of standard Fe_3_O_4_ crystals in accordance with JCPDS card number 19-0629. Diffraction peaks of the MNPs were observed at 2*θ* = 30.44°, 35.74°, 43.54°, 53.81°, 57.50°, and 63.04°, which corresponded to the (220), (311), (400), (422), (511), and (440) cubic spinel planes of Fe_3_O_4_, respectively. The characteristic peaks of MNP-βCD-TDI and IL-MNP-βCD-TDI, however, did not differ from the peaks of the MNPs, which thus confirmed that alteration of the surface of the MNPs did not affect their crystal phase. Furthermore, in terms of intensity, the decrease for MNP-βCD-TDI and the increase for IL-MNP-βCD-TDI are attributable to the presence of an amorphous layer of the βCD polymer, as well as the successful coating of the IL onto the surface of MNP-βCD-TDI.^58^

The thermal properties (Fig. 1B) of the MNPs were determined from the TGA curves, which indicated a three-stage degradation pattern. Basically, the first step of the degradation process (MNPs) corresponds to the loss of water at lower temperatures (30–160 °C). Next, the second stage of degradation involved both MNP-βCD-TDI and IL-MNP-βCD-TDI owing to the presence of carbamate linkages and βCD (250–350 °C), with a weight loss of 21%. The low weight loss shows that these adsorbents were thermally stable at high temperatures and the transformation of βCD into a polymeric form had increased its stability.^38,59^ The last stage of the degradation process occurred in the range of 350–600 °C and was perhaps due to the thermal decomposition of CD, which thus confirmed the grafting of the βCD polymer onto the surface of MNPs, with a weight loss of 19% for both materials. Apart from that, the weight loss (350–500 °C) for IL-MNP-βCD-TDI appears to be a result of thermal carbonisation and decomposition of organic groups, such as combustion of the IL.^58^ The trends in degradation and weight loss are detailed in Table 1(b).

The results of the SEM and TEM analyses are illustrated in Fig. S1 (ESI S1†). The SEM analysis was performed to determine the surface morphologies of the adsorbents. The micrograph that was obtained displayed several similarities in the morphologies because the adsorbents were generally synthesised from the same base, which was magnetic. Fig. S1(a)† shows the surface morphology of the synthesised MNPs, whereas Fig. S1(b)† shows the chemical coating of the βCD polymer onto the surface of MNPs. The polymerization process with TDI led to a morphology that resembled a conglomerate of beads with small cavities surrounding the MNPs. Furthermore, upon the loading of the IL, as shown in Fig. S1(c),† the presence of the IL is clearly indicated on the surface of MNP-βCD-TDI (whitish appearance).

TEM was performed for two primary purposes: (1) to exemplify the core–shell structure of the adsorbents and (2) to confirm the spherical morphology of the agglomerates, as illustrated in Fig. S1(a′)–(c′).† The average diameter of the bare MNPs was approximately 14 nm but increased slightly to 16 nm after coating with the βCD polymer. Subsequently, the presence of the IL on the surface of MNP-βCD-TDI resulted in well-dispersed absorbance and an increase in the particle diameter to 18 nm. Initially, the TEM images were analysed with ImageJ software in order to determine the average diameter from the particle distribution of the adsorbents. This step was carried out prior to the BET analysis so as to ascertain the accuracy of the analysis classification (microporous, mesoporous or macroporous). On the basis of the tabulated histogram data, the average diameter of the particles appeared to be between 2 nm and 6 nm, which corresponded to the mesoporous category (2–50 nm).

Next, Table 1(c) presents the findings obtained from the BET analyses that were performed for all the adsorbents. The surface area of the bare MNPs was 90.14 m^2^ g^−1^ and decreased to 54.72 m^2^ g^−1^ after they were coated with the polymeric adsorbent. Upon the introduction of the IL, the surface area of the adsorbent decreased to 42.95 m^2^ g^−1^, which appeared to be an effect of the coating of the MNPs. In addition, the Barrett–Joyner–Halenda (BJH) model was employed in this study to calculate the pore size distribution of the adsorbents. The average pore sizes of these adsorbents were 14.72 nm for MNPs, 15.49 nm for MNP-βCD-TDI, and up to 18.27 nm upon the loading of the IL, which hence indicated the mesoporous type of adsorbent owing to a decrease in pore volume from 0.33 cm^3^ g^−1^ to 0.21 cm^3^ g^−1^ and 0.19 cm^3^ g^−1^, respectively. Such findings, nevertheless, appear to contradict those of a prior study, in which MNPs coated with βCD and functionalized with an IL for a sensor application displayed increases in both surface area and pore volume.^52^ Such a contrast may be attributed to the unique properties of CD and the IL. Furthermore, the N_2_ adsorption/desorption isotherms showed that all the adsorbents exhibited typical type IV isotherms, which correspond to the mesoporous type of material, with H3-type hysteresis loops, which relate to non-rigid aggregates of plate-like particles, as portrayed in Fig. 1C.^58^

Next, the results obtained from the CHN analyses are tabulated in Table 1(d). The elemental compositions of the adsorbents were determined by a CHN analyser. As a result, the CHN findings confirmed the successful coating of the βCD polymer on the surface of the MNPs, with increases in the following contents: 18.96% of C, 1.92% of H, and 3.79% of N from the low amounts in the MNPs, which were 0.23% of both C and H and 0.05% of N. As indicated in the table, the nitrogen content rose to 9.32% owing to the presence of the IL.^60^ This demonstrates that the loading of the IL onto the surface of MNP-βCD-TDI was indeed a success, together with increases in elemental percentages to 47.75% of C and 5.28% of H.

### 3.2 Optimisation of batch experiments

#### 3.2.1 Preliminary batch study

The results presented in Fig. 1D show the efficiency of the adsorption of the three selected parabens (PP, BP, and ArP) by the synthesised materials. As the primary focus of this study is on the newly developed materials, the results appear to indicate a significant increase in adsorption efficiency for (c) in comparison with (a) and (b). Moreover, the presence of the IL in (c) greatly increased the selectivity towards parabens owing to various factors, such as hydrophobic interactions between βCD and those parabens, including π–π interactions and hydrogen bonding between the aromatic rings in the parabens and the imidazolium ring in IL-MNP-βCD-TDI. In addition, the performance of this material was further investigated and the formation of the inclusion complex between βCD and one selected paraben (ArP) was analysed.

#### 3.2.2 Effect of solution pH

**Fig. 2:**
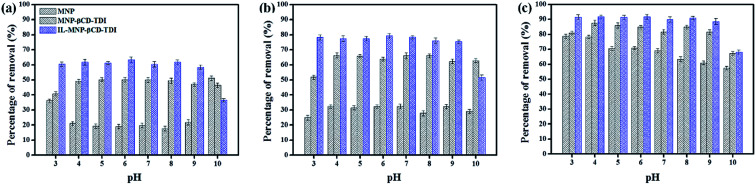
Effect of initial pH on the adsorption of (a) PP, (b) BP, and (c) ArP using three different types of materials (conditions: sorbent, 20 mg; initial concentration, 10 mg L^−1^; adsorption time, 120 min; temperature, 298 K).

The effect of pH values that ranged from 3 to 10 on the adsorption efficiency of the three types of adsorbents for the selected parabens (PP, BP, and ArP) is illustrated in Fig. 2. The findings indicate that the sorption was pH-dependent and a pH of 6 was optimal for all the studied parabens. The optimum pH determined from the graphs indicates the adsorption efficiency of the MNPs, which was 19.10%, 32.22%, and 70.80% for PP, BP, and ArP, respectively, whereas the values for MNP-βCD-TDI exhibited an increase as follows: 50.19%, 63.85%, and 84.76%, respectively. Upon the loading of the IL, the percentage removal for IL-MNP-βCD-TDI significantly increased to 60.37%, 79.31%, and 91.69% for PP, BP, and ArP, respectively, which was mainly due to the presence of the IL, which enhanced the hydrophobicity of the polymer.^40^ Besides, this could also be due to the presence of more active sites in IL-MNP-βCD-TDI, as well as the physical interactions between the adsorbent and the adsorbates dominated by van der Waals forces, formation of inclusion complexes, and hydrogen bonding.^29,37^ In this study, the formation of inclusion complexes seems to emerge as the main interaction, because the cavity of βCD was retained throughout polymerisation and coating of the polymer onto the surface of MNPs. Moreover, the π–π interaction between the imidazolium ring in the IL and all the studied parabens may also be a reason for the higher removal efficiency of IL-MNP-βCD-TDI.

After comparing the types of adsorbents, IL-MNP-βCD-TDI was selected as the adsorbent for this study. As evidenced in Fig. 2, the adsorption efficiency displayed by IL-MNP-βCD-TDI increased but appeared to exhibit slightly similar trends for all the studied parabens (PP, BP, and ArP) from a pH of 3 to a pH of 6. The optimum pH refers to a pH of 6, and a decline in adsorption efficiency was observed from a pH of 7 to a pH of 10. In acidic conditions (pH < 6), the amount of protons was maximized owing to protonation of the remaining hydroxyl groups on the surface of MNPs, which may saturate the sorbent sites and thus generate a more cationic surface of IL-MNP-βCD-TDI.^4,58^ In addition, the parabens were also in their protonated form as they only participate in π–π interaction and electrostatic repulsion. At a pH of 6, nonetheless, the paraben compounds began to adopt their neutral forms, which hence facilitated their participation in hydrophobic interactions with the βCD polymer cavity, whereas the presence of imidazole rings in the IL suggests π–π interactions and hydrogen bonding. In basic conditions (pH > 6), deprotonation of residual hydroxyl groups on MNPs could make the surface of the adsorbent anionic and hence decrease the hydrophobicity of the adsorbent. Because the p*K*_a_ values of the parabens are approximately 8.3, they also exist in the anionic form, which thus results in fewer interactions between the targeted analyte and the adsorbent. In brief, as the formation of an inclusion complex is deemed to be impossible with protonated and deprotonated parabens, a pH of 6 appears to be favourable because the parabens are neutral at this pH and can form inclusion complexes with the βCD cavity.

#### 3.2.3 Effect of contact time

**Fig. 3:**
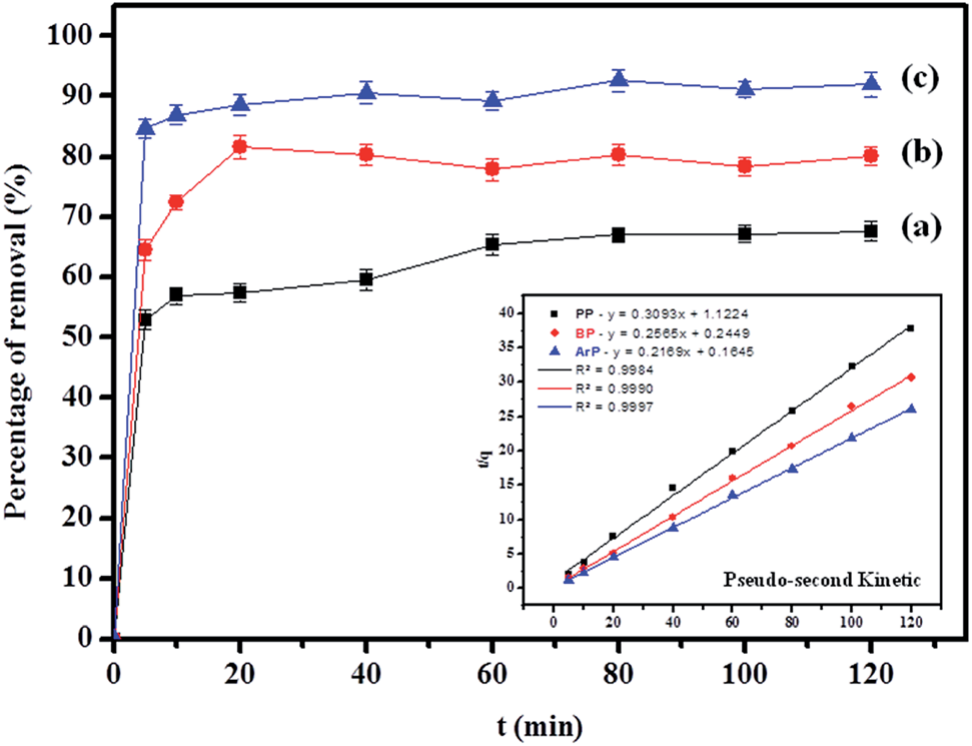
Effect of contact time on removal of (a) PP, (b) BP, and (c) ArP using IL-MNP-βCD-TDI (conditions: sorbent, 20 mg; initial concentration, 10 mg L^−1^; sample pH, 6; temperature, 298 K).

The effect of the contact time on the removal of the parabens (PP, BP, and ArP) by IL-MNP-βCD-TDI was thoroughly investigated within the time range of 0–120 min at room temperature (298 K), as illustrated in Fig. 3. The adsorption efficiency for the studied parabens seemed to be rapid during the first 5 min (ArP), 20 min (BP), and 40 min (PP), owing to the availability of additional active sites on the surface of IL-MNP-βCD-TDI. Nevertheless, the time taken for the parabens to interact with these active sites strongly depended on the alkyl chains present within the parabens. Therefore, the longer was the alkyl chain (ArP > BP > PP), the longer was the time taken by the parabens to saturate the active sites. Besides, the adsorption process was carried out up to 120 min, whereas the optimum contact time for all the studied parabens was 80 min, with efficiencies of 67.03%, 80.33%, and 92.61% for PP, BP, and ArP, respectively. After 80 min, the adsorption rate was observed to be constant. Hence, 80 min was selected for all the studied parabens as the equilibrium point throughout the study.

#### 3.2.4 Effect of initial concentration

**Fig. 4:**
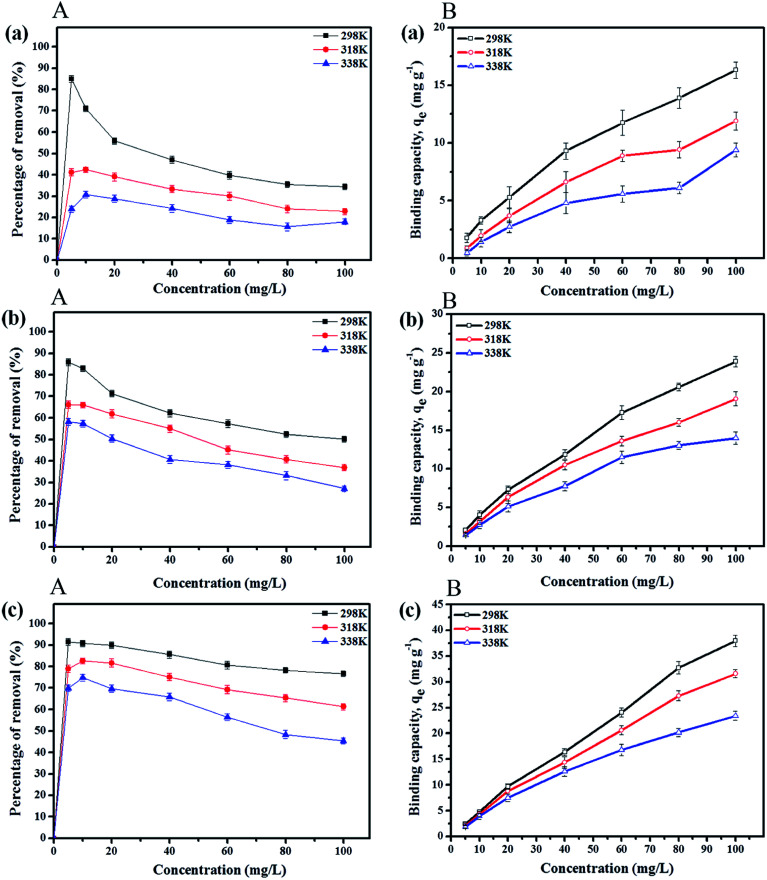
Effects of initial concentration (A) and solution temperature (B) on removal of (a) PP, (b) BP, and (c) ArP using IL-MNP-βCD-TDI at various temperatures (conditions: sorbent, 20 mg; adsorption time, 80 min; sample pH, 6).

Adsorption experiments at initial paraben concentrations of 5 to 120 mg L^−1^ were performed by maintaining the amount of the adsorbent (IL-MNP-βCD-TDI) at 20 mg at the optimum pH for 80 min at various temperatures, namely, 298 K, 318 K, and 338 K, for which the results are presented in Fig. 4A. The findings indicate that the percentage removal for all the studied parabens (ArP > BP > PP) decreased with an increase in the initial concentration. This can be explained by the fact that the surface of the adsorbent has a limited number of active sites, which suggests that saturation of the paraben compounds occurs above certain concentrations. Besides, the temperature also greatly affected the removal of all the studied parabens, as a rise in temperature reduced the rate of removal, which was perhaps due to degradation of the parabens at higher temperatures. Thus, the percentage removal reached equilibrium at 80 mg L^−1^ at room temperature (298 K).

#### 3.2.5 Effect of solution temperature

The temperature also influenced the adsorption capacities *q*_e_ (mg g^−1^) for the paraben compounds (PP, BP, and ArP), as illustrated in Fig. 4B. The graph portrays the trends displayed in the adsorption capacity for all the studied parabens, which seemed to decrease with an increase in temperature. Moreover, insignificant variations were discovered in the adsorption capacities at all the studied temperatures (298 K, 318 K, and 338 K) at lower concentrations (5 and 10 mg L^−1^). Nevertheless, the adsorption capacities appeared to be optimal at room temperature (298 K) at higher concentrations (80 and 100 mg L^−1^). These results indicate the exothermic nature of the adsorption process for all the studied parabens, which was largely due to the tendency of the parabens towards desorption from the surface of the adsorbent (IL-MNP-βCD-TDI).^61^ Hence, 298 K is the most suitable temperature for the removal of all the studied parabens using IL-MNP-βCD-TDI.

#### 3.2.6 Effect of sorbent dosage

**Fig. 5:**
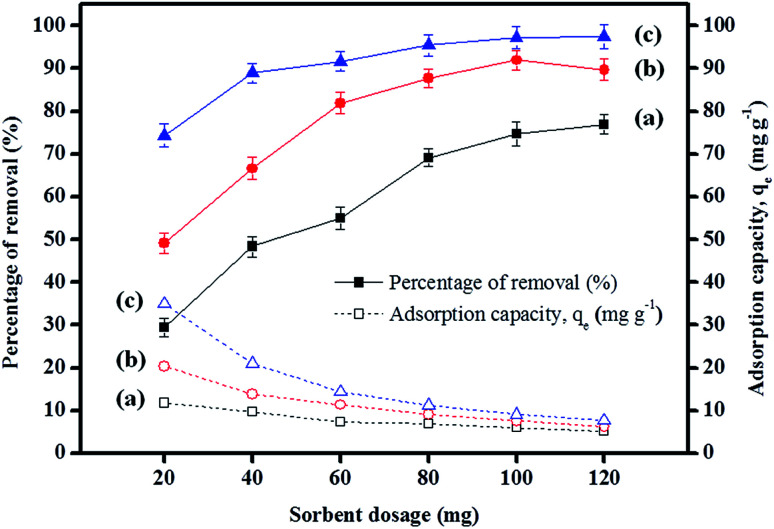
Effect of sorbent dosage on the adsorption of (a) PP, (b) BP, and (c) ArP (conditions: initial concentration, 80 mg L^−1^; adsorption time, 80 min; temperature, 298 K).

The results depicted in Fig. 5 indicate that the increase in the percentage removal is attributable to an increase in sorbent dosage up to 100 mg, but this was almost unchanged after 100 mg. This was mainly due to the increase in the number of active sites and surface areas during the adsorption process.^62^ Besides, the adsorption capacities displayed by IL-MNP-βCD-TDI for all the studied parabens decreased as the dosage was increased. This signifies that all the active sites were entirely exposed and saturated at a lower dosage, whereas only part of the active sites were utilized at a higher dosage owing to unsaturation of the adsorption sites *via* the adsorption reaction.^63^

### 3.3 Adsorption study

The adsorption process refers to the adhesion of elements to a surface that involves physisorption, chemisorption or electrostatic attraction.^64^ This method enhances water quality by removing toxic organic pollutants or chemical species, including organic compounds and trace metals.^37,65^

#### 3.3.1 Adsorption kinetics

In order to determine the adsorption mechanism, the kinetic parameters were calculated *via* the adsorption of PP, BP, and ArP on IL-MNP-βCD-TDI using the (a) pseudo-first-order,^66^ (b) pseudo-second-order,^67,68^ (c) Elovich,^69^ and (d) intraparticle diffusion^70^ models. Hence, to describe the validity and suitability of the adsorption kinetics, the normalized standard deviation Δ*q* (%) and relative error (%) were calculated using the following equations:3
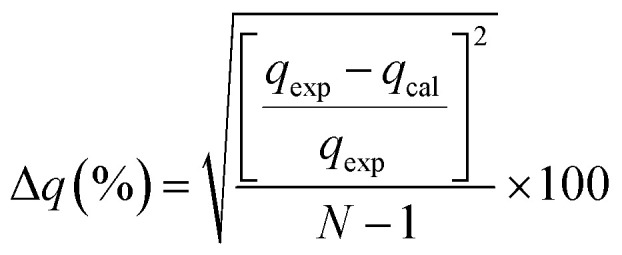
4
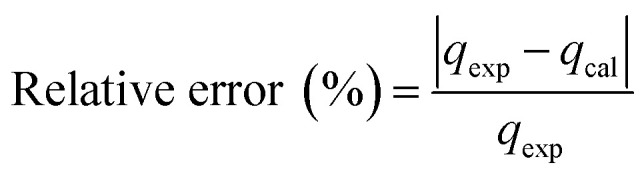
where *N* represents the number of data points, whereas *q*_exp_ and *q*_cal_ (mg g^−1^) refer to the experimental and calculated adsorption capacities. According to the theory, a model fits better if the values of Δ*q* and the relative error (%) are lower.^69^Table 2 presents the fitting results for the adsorption of PP, BP, and ArP on IL-MNP-βCD-TDI, including the goodness and accuracy of the studied models, which were compared quantitatively in terms of the coefficients of determination (*R*^2^), normalized standard deviations, Δ*q* (%), and relative errors (%).

**Table 2:** Kinetic parameters for adsorption of PP, BP, and ArP on IL-MNP-βCD-TDI

Kinetic model	Parameter	IL-MNP-βCD-TDI		
		PP	BP	ArP
Pseudo-first-order	*q*_e,exp_ (mg g^−1^)	3.0990	3.8721	4.6271
	*q*_e,cal_ (mg g^−1^)	0.6295	0.2883	0.0812
	*k*_1_ (min^−1^)	0.0343	0.0205	0.0177
	Δ*q* (%)	39.843	53.436	40.108
	Relative error (%)	79.687	92.554	98.245
	*R* ^2^	0.8510	0.8734	0.8609
Pseudo-second-order	*q*_e,cal_ (mg g^−1^)	3.2331	3.8986	4.6104
	*k*_2_ (min^−1^)	0.0852	0.2687	0.2860
	*H*	0.8906	4.0840	6.0792
	Δ*q* (%)	1.6355	0.2587	0.1364
	Relative error (%)	4.3272	0.6844	0.3609
	*R* ^2^	**0.9984**	**0.9990**	**0.9997**
Elovich	*q*_e,cal_ (mg g^−1^)	3.0836	3.8214	4.6245
	*A*	2.4621 × 10^3^	1.0289 × 10^5^	3.5744 × 10^11^
	*β*	4.4366	4.5579	7.1225
	Δ*q* (%)	0.2222	0.5856	0.0281
	Relative error (%)	0.4969	1.3094	0.0562
	*R* ^2^	**0.9969**	**0.9197**	**0.9993**
Intraparticle diffusion	*q*_e,cal_ (mg g^−1^)	3.0451	3.8285	4.5537
	*K* (mg g^−1^ min^−1^)	0.0832	0.0747	0.0376
	*C* (mg g^−1^)	2.3009	3.1604	4.2174
	Δ*q* (%)	0.6574	0.4597	0.5996
	Relative error (%)	1.7393	1.1260	1.5863
	*R* ^2^	0.9564	0.7364	0.8201

##### (a) Pseudo-first-order model

The Lagergren pseudo-first-order equation has been widely applied in the adsorption of various solutes from aqueous solutions onto solid adsorbents.^71^ Lagergren developed a kinetic equation that describes the adsorption of a liquid–solid system on the basis of the assumption that the rate of solute uptake over time is proportional to the variation in the saturation concentration and the amount of solid adsorbed over time.^72^ The equation is as follows:5
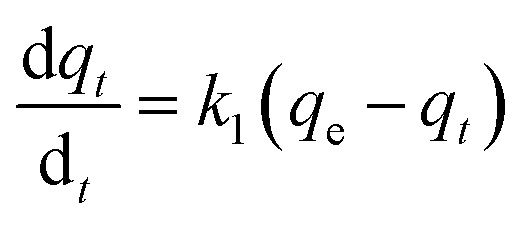
where *q*_*t*_ (mg g^−1^) and *q*_e_ (mg g^−1^) are the amounts of the analyte adsorbed on the synthesised adsorbent at time *t* (min) and equilibrium, whereas *k*_1_ (min^−1^) refers to the rate constant of pseudo-first-order adsorption. When *q*_*t*_ = 0 at *t* = 0, eqn (5) can be integrated to give the following equations:6
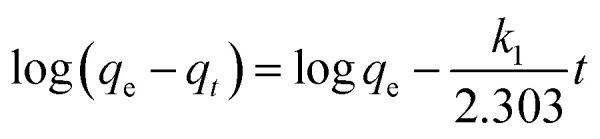

7 ln(*q*_e_ − *q*_*t*_) = log *q*_e_ − *k*_1_*t*

If the experimental data fit this kinetic model, a straight line can be obtained on a graph of ln(*q*_e_ − *q*_*t*_) *versus t*, and thus adsorption is followed by diffusion through a boundary.^66^ Besides, if the data fail to fit this equation, diffusion is excluded as the rate-determining step. The linear plot has *k*_1_ as the slope, whereas log *q*_e_ is the intercept.

##### (b) Pseudo-second-order model

The behaviour of a wide range of adsorption processes follows the pseudo-second-order model, which has chemisorption as the rate-determining step and adheres to the assumption that the adsorption mechanism relies on both the adsorbate and the adsorbent.^66,68^ According to Ho and McKay, if adsorption is in accordance with the pseudo-second-order model, the following equation gives the rate law:^73^8
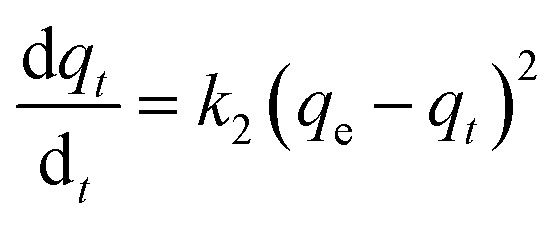


Upon integrating eqn (8) with *q*_*t*_ = 0 at *t* = 0, *k*_2_ (g mg^−1^ min^−1^) is the equilibrium rate constant of the pseudo-second-order model, whereby the equation that is obtained can be rearranged to give:9
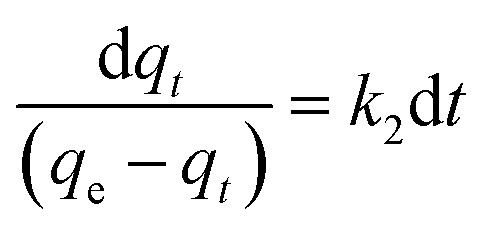


By integrating eqn (9) for values of *t* = *t* and *q*_*t*_ = *q*_*t*_, the integrated rate law is as follows:10
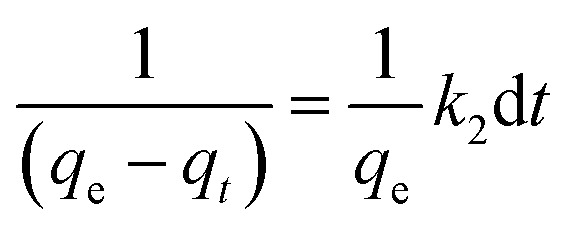


Moreover, eqn (10) can be rearranged into the following linear form:11
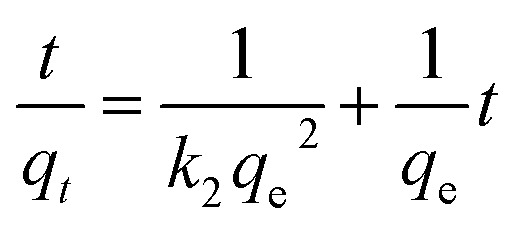
from which the initial adsorption rate (mg g^−1^ min^−1^) is *h* = *k*_2_*q*_e_^2^. A plot of 
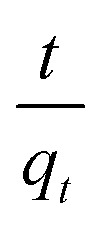
*versus t* can be generated in which the values of *q*_e_, *k*_2_, and *t*_1/2_ are embedded; hence, a linear plot of 
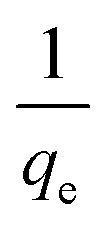
*versus*
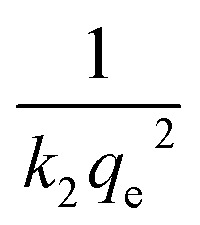
 gives the slope and intercept of the pseudo-second-order model.

Table 2 shows that the pseudo-second-order model appears to fit the adsorption mechanism for all the paraben compounds more closely in comparison with the other studied models, as shown by its excellent coefficient of determination, *R*^2^ > 0.998 (Fig. 3), and lower Δ*q* values of 1.6355% (PP), 0.2587% (BP), and 0.1364% (ArP), with relative errors of 4.3272% (PP), 0.6844% (BP), and 0.3609% (ArP), respectively. In fact, these results can be confirmed by the closeness of the *q*_cal_ and *q*_exp_ values in the pseudo-second-order model. In particular, the adsorption process undertaken in this study for all the parabens was controlled by chemisorption,^74^ which involves valence sharing or the exchange of electrons^37,75^ and is based on the assumption that the adsorption mechanism depends on both the adsorbate and the adsorbent.^66,68^ Besides, the pseudo-second-order adsorption rate constants of the paraben compounds (*k*_2_) and the initial sorption rates (*h*) on IL-MNP-βCD-TDI are in the order of ArP > BP > PP.

##### (c) Elovich model

In an attempt to prove that the adsorption process was indeed based on chemisorption, the Elovich model was incorporated in this study. The Elovich equation is expressed as follows:^69^12
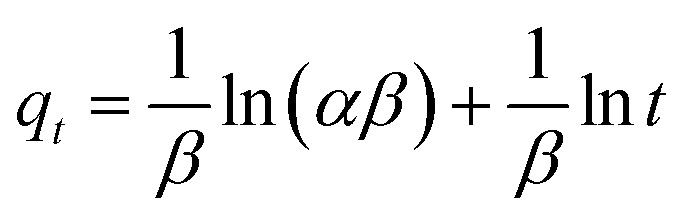
where *α* is the initial sorption rate (mg g^−1^ min^−1^), whereas *β* denotes the extended surface coverage and the activation energy for chemisorption (g mg^−1^). A linear plot of *q*_t_*versus* ln *t* has 
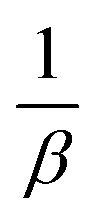
 and 
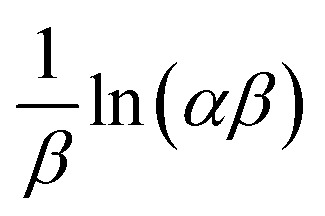
 as the slope and intercept, respectively.

The exceptional coefficient of determination, *R*^2^ > 0.9, seemed to fit a linear Elovich plot well. Moreover, in determining the occurrence of chemisorption, the Δ*q* values and relative errors appeared to be lower, which indicated a difference in values between *q*_cal_ and *q*_exp_. The values of *β*, on the other hand, signify the number of sites available for the adsorption process. Furthermore, *α* represents the adsorbed quantity when ln *t* = 0, *i.e.*, when *t* is 1 min.^76^ Hence, the models fit the kinetic data for the adsorption of all the studied parabens in the following order: pseudo-second-order > Elovich > pseudo-first-order kinetics.

##### (d) Weber–Morris model

**Fig. 6:**
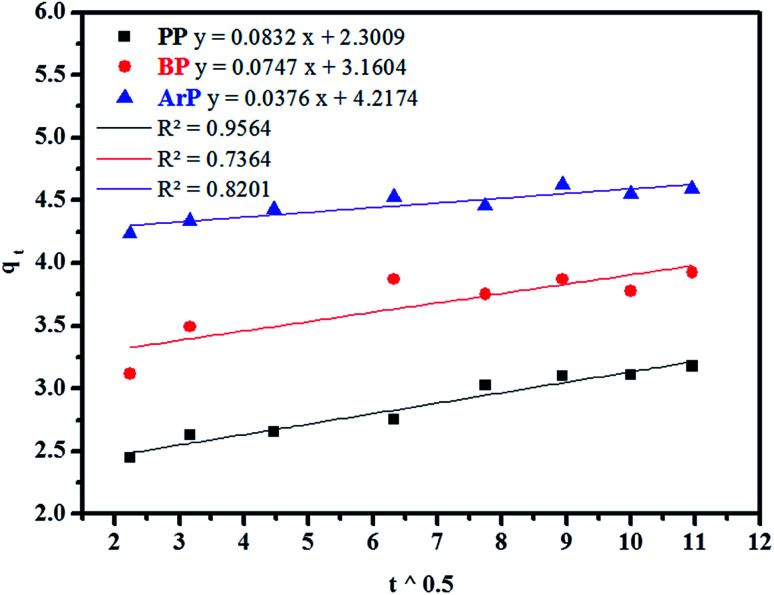
Kinetic intraparticle diffusion model for the adsorption of PP, BP, and ArP on IL-MNP-βCD-TDI.

Weber–Morris intraparticle diffusion is defined by the equation below:

13 *q*_*t*_ = *Kt*^0.5^ + *c*

where *K* refers to the intraparticle diffusion rate constant (mg g^−1^ min^1/2^) and *c* represents the intercept (mg g^−1^). The parameters *K* and *c* are determined from a linear plot of *q*_*t*_*versus t*^1/2^. From the data presented in Fig. 6, the line does not pass through the origin, which thus points out that intraparticle diffusion is not the rate-determining step but is indicative of some form of boundary layer within which another kinetic model that describes the adsorption rate may operate simultaneously.^70^ The value of *c* suggests the boundary layer thickness that corresponds to a better adsorption mechanism for all the studied parabens. The greater is the intercept, for example, 2.3009 mg g^−1^ (PP), 3.1604 mg g^−1^ (BP), and 4.2174 mg g^−1^ (ArP), the greater is the boundary layer effect.

#### 3.3.2 Adsorption isotherms

Adsorption isotherms describe the equilibrium adsorption of an adsorbate on an adsorbent's surface at a given constant temperature and pH. Specifically, an isotherm is represented by plotting the equilibrium concentration of a compound on the adsorbent as a function of its equilibrium concentration in solution.^77^ In brief, an adsorption isotherm represents the interaction between the adsorbate and the adsorbent. Over the years, many equilibrium isotherm models have been designed to study the mechanism of these interactions. As such, this study incorporated three main isotherms, namely, the (a) Langmuir, (b) Freundlich, and (c) Temkin models. Non-linear equations have to be transformed into equations that are linear so as to obtain a linear fitting value of *R*^2^. The *R*^2^ value that is closest to unity indicates that an isotherm model provides the best fit to the experimental data. The experimental equilibrium data for the adsorption of PP, BP, and ArP on IL-MNP-βCD-TDI under the best conditions at three different temperatures (298 K, 318 K, and 33 8 K) are tabulated in Table 3.

**Table 3:** Details of isotherm constants for various adsorption isotherms for the adsorption of PP, BP, and ArP on IL-MNP-βCD-TDI

Analyte	Isotherm model			
	Parameter	IL-MNP-βCD-TDI		
		298 K	318 K	338 K
**Langmuir**				
PP	*q*_m_ (mg g^−1^)	18.48	18.12	15.63
	*b* (L mg^−1^)	0.0658	0.0216	0.0130
	*R*_L_	0.1627	0.3719	0.4959
	*R* ^2^	0.9512	0.9770	0.7522
BP	*q*_m_ (mg g^−1^)	28.49	25.13	19.08
	*b* (L mg^−1^)	0.0717	0.0414	0.0376
	*R*_L_	0.1505	0.2348	0.2526
	*R* ^2^	0.9589	0.9898	0.9870
ArP	*q*_m_ (mg g^−1^)	50.25	46.73	31.55
	*b* (L mg^−1^)	0.1004	0.0490	0.0463
	*R*_L_	0.1065	0.1963	0.2054
	*R* ^2^	0.9801	0.9709	0.9850

**Freundlich**				
PP	*q*_m_ (mg g^−1^)	17.03	13.26	8.92
	*K*_F_ [(mg g^−1^) (L mg^−1^)^1/*n*_F_^]	2.1145	0.5338	0.2689
	*n*_F_	2.1004	1.3641	1.2514
	1/*n*_F_	0.4761	0.7331	0.7991
	*R* ^2^	**0.9966**	0.9809	0.9572
BP	*q*_m_ (mg g^−1^)	30.86	24.42	17.02
	*K*_F_ [(mg g^−1^) (L mg^−1^)^1/*n*_F_^]	2.7714	1.3993	1.0464
	*n*_F_	1.8182	1.5326	1.5711
	1/*n*_F_	0.5500	0.6525	0.6365
	*R* ^2^	**0.9955**	0.9781	0.9829
ArP	*q*_m_ (mg g^−1^)	87.67	59.05	34.06
	*K*_F_ [(mg g^−1^) (L mg^−1^)^1/*n*_F_^]	4.8239	2.5734	1.8256
	*n*_F_	1.5110	1.3986	1.4975
	1/*n*_F_	0.6618	0.7150	0.6678
	*R* ^2^	**0.9865**	0.9700	0.9606

**Temkin**				
PP	*B*	3.0564	3.1254	2.3505
	*K*_T_ (L mg^−1^)	1.4711	0.3877	0.2954
	*b*_T_ (kJ mol^−1^)	810.62	845.92	1195.5
	*R* ^2^	0.9074	0.9605	0.9072
BP	*B*	4.9155	4.6864	3.6612
	*K*_T_ (L mg^−1^)	1.3806	0.6428	0.5440
	*b*_T_ (kJ mol^−1^)	504.03	564.15	767.54
	*R* ^2^	0.9283	0.9731	0.9642
ArP	*B*	8.7073	8.0738	5.9534
	*K*_T_ (L mg^−1^)	1.8765	0.8944	0.7054
	*b*_T_ (kJ mol^−1^)	284.54	327.46	472.02
	*R* ^2^	0.9455	0.9648	0.9833

##### (a) Langmuir model

The Langmuir isotherm has been widely used to calculate the adsorption capacity for pollutants. Langmuir developed an equilibrium isotherm to reveal the correlation between the amount of gas adsorbed on the surface and the pressure of the gas.^78^ According to Langmuir, several assumptions that apply to this model are: (1) adsorption only takes place at specific homogeneous sites within the adsorbent, *i.e.*, all surface adsorption sites have equal adsorption energies; (2) in adsorption, there are a finite adsorption capacity and a saturation point; and (3) adsorption of an adsorbate is directly proportional to its concentration in solution. The linear form of the Langmuir isotherm equation is represented as follows:14
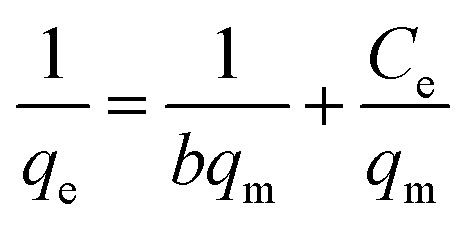
where *C*_e_ (mg L^−1^) is the equilibrium concentration of the adsorbate, *q*_e_ (mg g^−1^) refers to the adsorption capacity at equilibrium, and *q*_m_ (mg g^−1^) and *b* (L mg^−1^) are the Langmuir constants related to the adsorption capacity and rate of adsorption, respectively. Besides, *q*_m_ and *b* can be determined from a linear plot of *C*_e_/*q*_e_*versus C*_e_, which has a slope of 
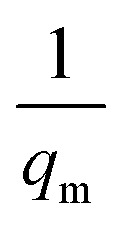
 and an intercept of 
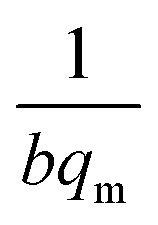
. Moreover, the dimensionless separation factor (*R*_L_) can be calculated to determine if the adsorption process is favourable, as shown in the equation below, where *C*_o_ (mg L^−1^) represents the initial capacity for the adsorbate at equilibrium:15
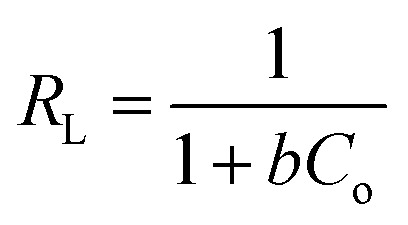
*R*_L_ = 0 for irreversible adsorption, 0 < *R*_L_ < 1 for favourable adsorption, and *R*_L_ = 1 for linear adsorption, whereas *R*_L_ > 1 for unfavourable equilibrium.

Basically, the Langmuir model is applied to monolayer adsorption on a homogeneous system. As for this study, the adsorption process is assumed to follow the Langmuir model because the model appears to fit the experimental data rather well with exceptional coefficients of determination (*R*^2^ > 0.95) for all the studied parabens, except for PP at 338 K, which gave a value of 0.7522. This result reflects the lack of hydrophobicity in PP, and hence its interaction with the βCD cavity was disregarded, primarily owing to the higher temperature (338 K), at which the analyte failed to remain in solution any longer and was subjected to degradation. The maximum monolayer adsorption (*q*_m_) was 50.251 mg g^−1^ for the adsorption of ArP, with an *R*_L_ value of 0.1065 at 298 K. The *R*_L_ values that were obtained for all the studied parabens seem favourable at all the studied temperatures as they exceeded zero but were less than unity (0 < *R*_L_ < 1).

##### (b) Freundlich model

The correlation between the adsorbate and the adsorbent at equilibrium was estimated from the coefficient of determination (*R*^2^), which indicated a better linear form that fitted the Freundlich isotherm model, followed by the Langmuir isotherm model. In fact, the Freundlich equation describes the adsorption equilibrium. This model is employed for adsorption on heterogeneous surfaces with various classes of adsorption sites, together with various energy levels of adsorption. The Freundlich equation is expressed as follows:^73^16
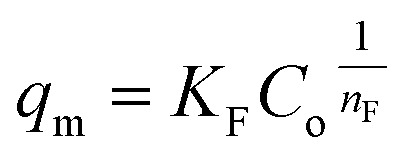
where *q*_m_ is the equilibrium concentration of the adsorbate on the adsorbent (mg g^−1^), *C*_o_ refers to the initial equilibrium concentration of the adsorbate in solution (mg L^1^), *K*_F_ denotes the Freundlich constant associated with multilayer adsorption capacity, and 
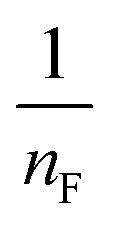
 represents the heterogeneity factor, where *n*_F_ is determined from the deviation from linear adsorption (adsorption is directly proportional to the concentration of the solution).^66^ From eqn (16), the following equation can be expressed in a linear form:17
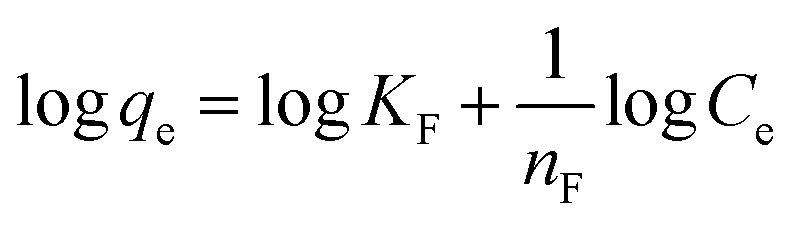


Thus, the experimental data plotted as log *q*_e_*versus* ln *C*_e_ were used to determine the intercept *K*_F_ and the slope 
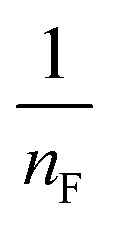
. The value of 
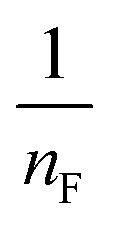
 suggests the process of the adsorption system.^79^ Besides, Tseng and Wu asserted that if *n*_F_ < 1 the process is chemical adsorption (unfavourable), whereas *n*_F_ = 1 indicates linear adsorption, and *n*_F_ > 1 refers to favourable physical adsorption.

The adsorption of PP, BP, and ArP on IL-MNP-βCD-TDI demonstrated that the *R*^2^ value in the Freundlich model exceeded 0.95 at all the studied temperatures. This implies that the adsorption process on IL-MNP-βCD-TDI involves a heterogeneous surface with many cavities (CD), isocyanate groups, and imidazolium rings.^37^ Moreover, *K*_F_ and *n*_F_ are the constants of the Freundlich isotherm that correspond to the adsorption capacity and intensity of the adsorbent, respectively. Hence, the significant decrease in the adsorption capacity (*K*_F_) for all the studied parabens as the temperature rose indicates that the adsorption was exothermic, *i.e.*, 2.1145–0.2689 (PP), 2.7714–1.0464 (BP), and 4.8239–1.8256 (ArP), as listed in Table 3. In addition, in order to prove that the adsorption process in this study is indeed favourable at lower temperatures, the intensity of the adsorbent (*n*_F_) was calculated from the Freundlich model, whereby the results appeared to be in the range of 1 < *n*_F_ < 10 for the adsorption of all the studied parabens.

On the other hand, the sorption of ArP seemed to be better than that of BP, followed by that of PP, on IL-MNP-βCD-TDI, and this phenomenon may be explained by the inclusion effect and the π–π interactions between the adsorbent and the adsorbates. Furthermore, the hydrophobicity of parabens depends on the alkyl chain in the parabens, whereby a longer alkyl chain results in higher hydrophobicity. Besides, the ability of βCD to form a stable inclusion complex relies on the hydrophobicity of the analyte, which resulted in higher affinity for ArP owing to its higher hydrophobicity in comparison with that of BP, followed by that of PP. In addition, π–π interactions occur *via* the imidazolium rings in the IL and the double bonds (aromatic rings) in the parabens. Because ArP possesses more double bonds, the interaction of ArP is stronger in comparison with that of BP, followed by that of PP. In brief, the order of the strength of the adsorption process on IL-MNP-βCD-TDI is ArP > BP > PP, which corresponds to the exothermic nature of the process (298 K > 318 K > 338 K).

##### (c) Temkin model

Temkin and Pyzhev incorporated the effect of some indirect and adsorbate/adsorbent interactions into the adsorption isotherm.^80^ It was also suggested that the heat of adsorption for all molecules in the adsorbent surface layer decreases linearly with the coverage. Adsorption is further characterized by a uniform distribution of binding energies up to the maximum binding energy. The Temkin isotherm can be expressed in a linear form, as given in the following equation:

18 *q*_e_ = *β* ln *KT* + *β* ln *C*_e_

where 
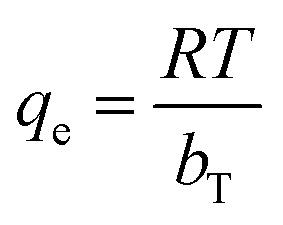
, *K*_T_ represents the equilibrium binding constant, which corresponds to the maximum binding energy (L mg^−1^), and *b*_T_ denotes the Temkin constant linked to the heat of adsorption. The constants *K*_T_ and *b*_T_ are determined from the intercept and slope, respectively, of a plot of *q*_e_*versus* ln *C*_e_.

The Temkin isotherm model also provides the best fit for the coefficient of determination (*R*^2^ > 0.90) for all the studied parabens at 298 K, 318 K, and 338 K. The Temkin constants *K*_T_ and *b*_T_ indicate the equilibrium binding constants that correspond to the maximum binding energy (L mg^−1^) and the heat of sorption (kJ mol^−1^), respectively. The heat of sorption increased as the temperature rose, from 810.62 to 1195.50 kJ mol^−1^ (PP), from 504.03 to 767.54 kJ mol^−1^ (BP), and from 284.54 to 472.02 kJ mol^−1^ (ArP).

Therefore, in order to confirm the applicability of the isotherm models and to obtain some specific indications of the adsorption behaviour, it is crucial to identify the values of the coefficient of determination (*R*^2^) that correspond to all three linear forms of the studied models. Hence, an inspection of the *R*^2^ values in Table 3 confirms that the equilibrium adsorption data display the best fit to the isotherm models in the order of Freundlich > Langmuir > Temkin for all the studied parabens (PP, BP, and ArP).

#### 3.3.3 Adsorption thermodynamics

**Fig. 7:**
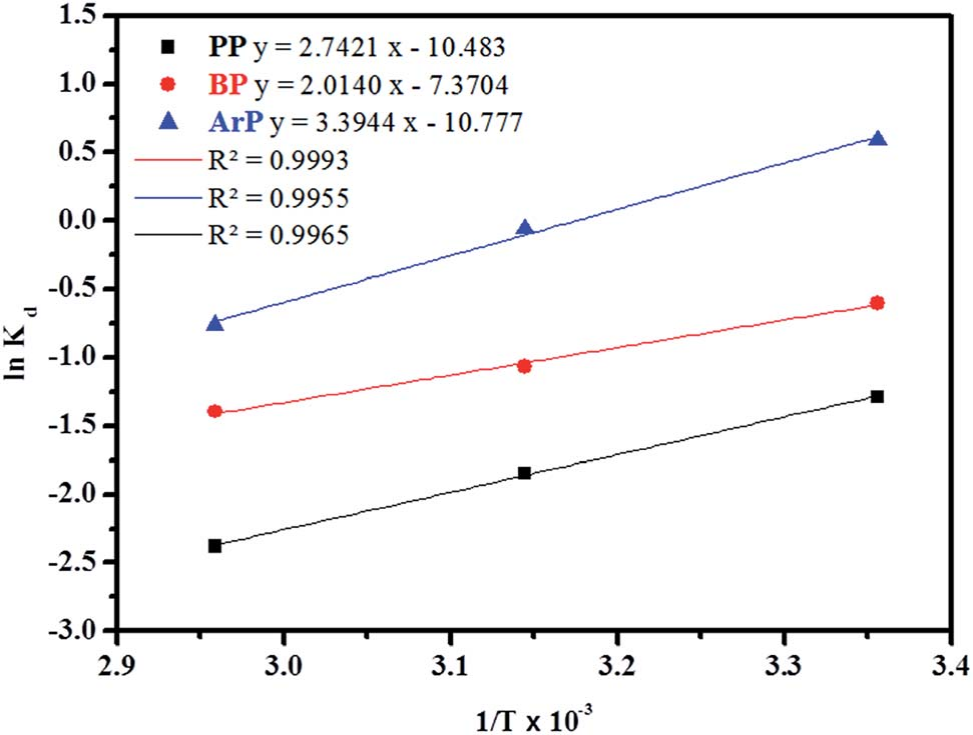
Thermodynamic plots for the adsorption of PP, BP, and ArP on IL-MNP-βCD-TDI.

In order to determine the thermodynamic feasibility of the adsorption of PP, BP, and ArP, the adsorption enthalpy (Δ*H*^o^), change in entropy (Δ*S*^o^), and Gibbs free energy (Δ*G*^o^) were determined from the slope and intercept of a van't Hoff plot of ln *K*_d_*versus* 1/*T* using eqn (20). In this case, *R* denotes the universal gas constant (kJ mol^−1^ K^−1^) and *T* is the temperature (K), whereas *K*_d_ refers to the equilibrium constant (m^3^ mol^−1^), which is determined from eqn (21), of which the results are tabulated in Table 4, whereas Fig. 7 presents the thermodynamic plots (van't Hoff plots) for the adsorption process.

19 Δ*G*^o^ = −*RT* ln *K*_d_

20
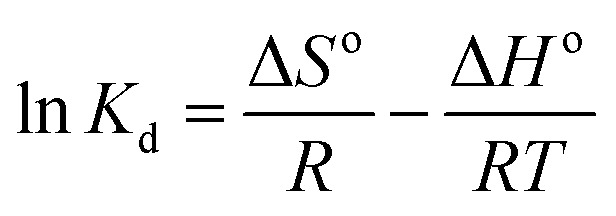
21
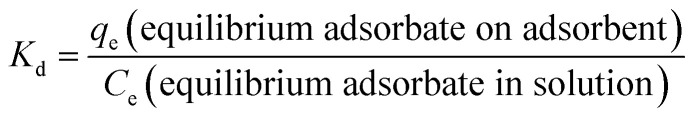


**Table 4:** Thermodynamic parameters for all the studied parabens

Analyte	*T* (K)	Δ*G*^o^ (kJ mol^−1^)	Δ*H*^o^ (J mol^−1^)	Δ*S*^o^ (J K^−1^ mol^−1^)
PP	298	25.95	−22.80	−87.16
	318	27.70		
	338	29.44		
BP	298	18.24	−16.74	−61.28
	318	19.47		
	338	20.70		
ArP	298	26.67	−28.22	−89.60
	318	28.46		
	338	30.26		

From the van't Hoff plots, Δ*H*^o^ was found to be negative (−22.80, −16.74, and −28.22 J mol^−1^ for PP, BP, and ArP, respectively), which confirms that the adsorption mechanism is indeed exothermic. Moreover, the negative values of Δ*S*^o^ for all the studied parabens (−87.17, −61.28, and −89.60 J K^−1^ mol^−1^, respectively) are attributed to a decrease in the degree of freedom at the solid/solution interface in the adsorption process.^81,82^ Specifically, in order to confirm the spontaneity of the adsorption process, Δ*G*^o^ should have a negative value at a given temperature. Nevertheless, the thermodynamic parameters in this study appeared to be positive but low in terms of the value of Δ*G*^o^ and thus indicated a feasible process that is non-spontaneous.^83^ Hence, the adsorption process for all the studied parabens can be concluded to be exothermic in nature and feasible, and could be spontaneous if the temperature is sufficiently low.

### 3.4 Analysis of real samples

**Fig. 8:**
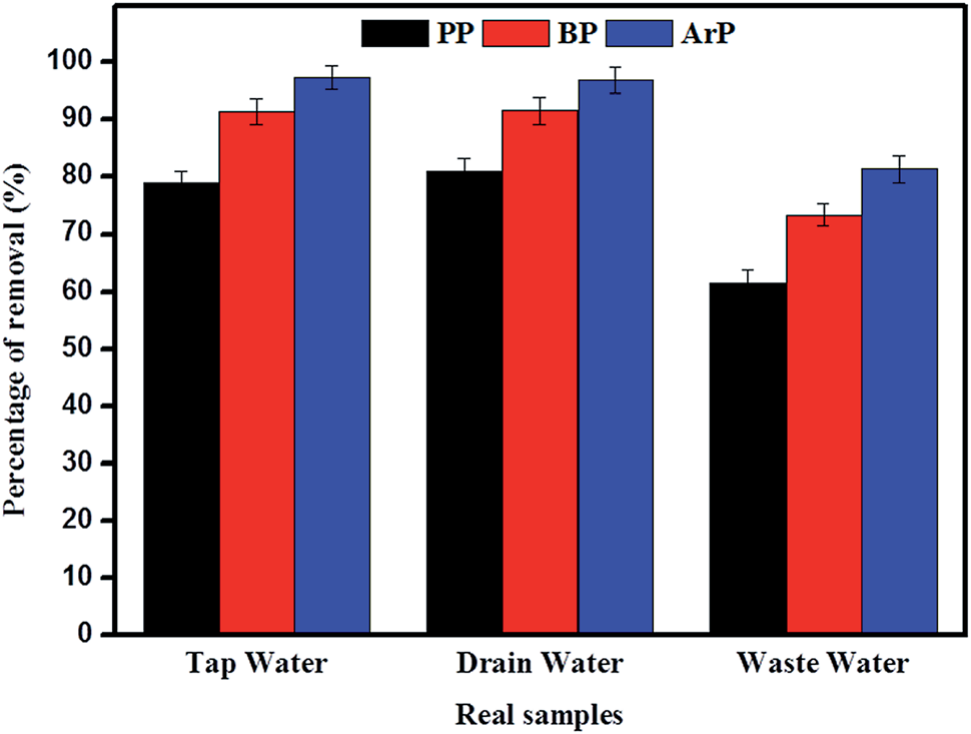
Analysis of removal of PP, BP, and ArP from real samples (conditions: sorbent, 100 mg; initial concentration, 80 mg L^−1^; adsorption time, 80 min; temperature, 298 K).

The adsorption of PP, BP, and ArP was further investigated using real water samples. As illustrated in Fig. 8, the results for tap water and drain water appeared to be good enough in terms of removal of all the studied parabens. However, a decrease in the percentage removal was noted for industrial wastewater, which was mainly due to the matrix effect of the water itself. Moreover, industrial wastewater is known to contain many types of contaminants, which results in strong competition between unknown analytes and the targeted analytes to saturate the surface of the materials. Therefore, the low removal efficiency for the industrial wastewater sample is no surprise, although the results are still above the acceptable levels of 60%, 70%, and 80% for PP, BP, and ArP, respectively.

### 3.5 Regeneration of adsorption activity displayed by IL-MNP-βCD-TDI

**Fig. 9:**
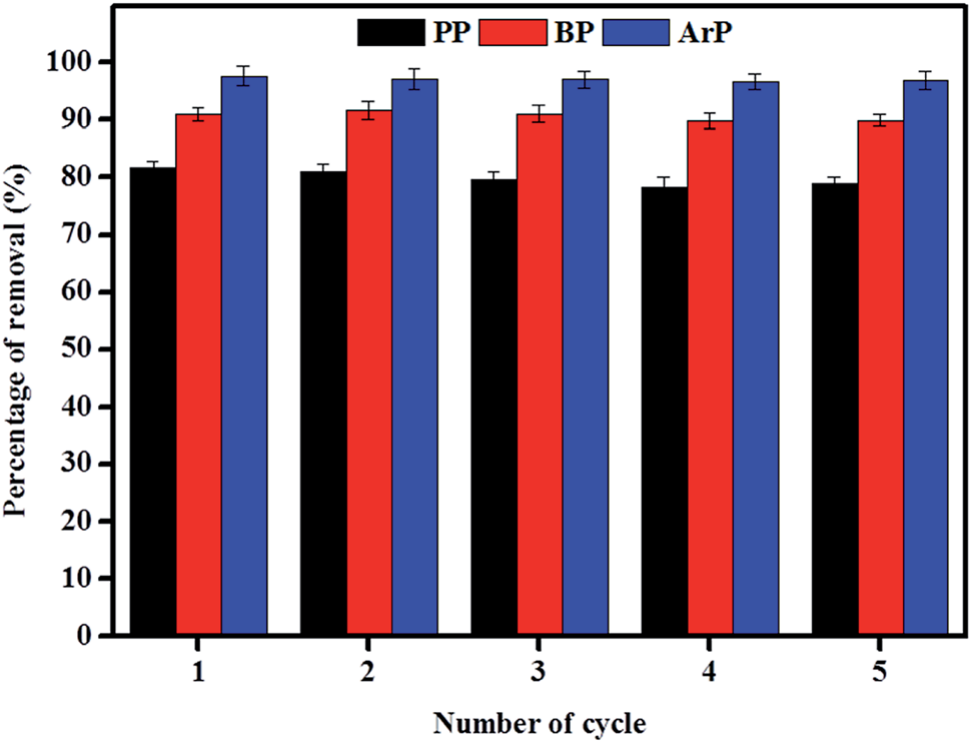
Reusability of IL-MNP-βCD-TDI for the adsorption of PP, BP, and ArP (conditions: sorbent, 100 mg; initial concentration, 80 mg L^−1^; adsorption time, 80 min; temperature, 298 K; desorption solvent, 5 mL DI and 5 mL ACN by vortexing).

The reuse of adsorbents has become an area of interest among many researchers, and this study area must be taken into consideration owing to its vast range of applications. Therefore, in order to investigate the possibility of the reuse of the material examined in this study, sequential adsorption–desorption experiments in batch mode were performed over five cycles. From the data presented in Fig. 9, an insignificant reduction in removal efficiency was observed after the material had been reused five times. This result indicates that IL-MNP-βCD-TDI is indeed an exceptional and promising material that can be used to remove paraben compounds. The cycling tests were, nonetheless, discontinued in this particular study owing to the limited time available as a result of the time taken for the material to dry, as well as its reusability, which had been tested on three types of paraben compounds (PP, BP, and ArP).

### 3.6 Comparison of various magnetically based adsorbents

As listed in Table 5, various magnetically based adsorbents were compared with various types of analytes, primarily because adsorption studies relating to paraben compounds are rare. The only adsorption study, which was reported by Forte *et al.* (2015),^76^ suggested that polyacrylonitrile (PAN) beads have been used to remove methyl paraben, but these are not a magnetically based adsorbent. Therefore, a comparison of the sorbent dosage (mg), initial concentration (*C*_o_), equilibrium time, and maximum capacity (*q*_m_) was undertaken, and these parameters were compared with those for various magnetically based adsorbents. Because IL-MNP-βCD-TDI forms many types of interactions, a higher adsorption capacity was achieved by using a sorbent dosage of only 20 mg with an equilibrium time of 80 min.

**Table 5:** Comparison of removal studies using various MSPE-based adsorbents

Analytes	Adsorbent	Sorbent dosage (mg)	Initial concentration (mg L^−1^)	Reaction time (min)	pH	Maximum capacity, *q*_e_ (mg g^−1^)	Reference
DEHP	Fe_3_O_4_@P3TArH	10	15	120	7	52.63	6
Cu^2+^	CM-β-CD-Fe_3_O_4_	120	50–200	30	6	47.20	30
DNA	[C_6_MIM]-Fe_3_O_4_	15	20	10	3	19.80	54
Fluoride	Fe_3_O_4_@Al(OH)_3_ NPs	100	20	60	6.5	88.48	84
Reactive red 120 4-(2-pyridylazo)resorcinol	IL-Fe_3_O_4_	60	200	2	2.5	166.67	85
						49.26	
PP	IL-MNP-βCD-TDI	20	80	80	6	18.48	This work
BP						30.86	
ArP						87.67	

### 3.7 Adsorption behaviour of βCD–ArP *via* formation of inclusion complex

**Fig. 10:**
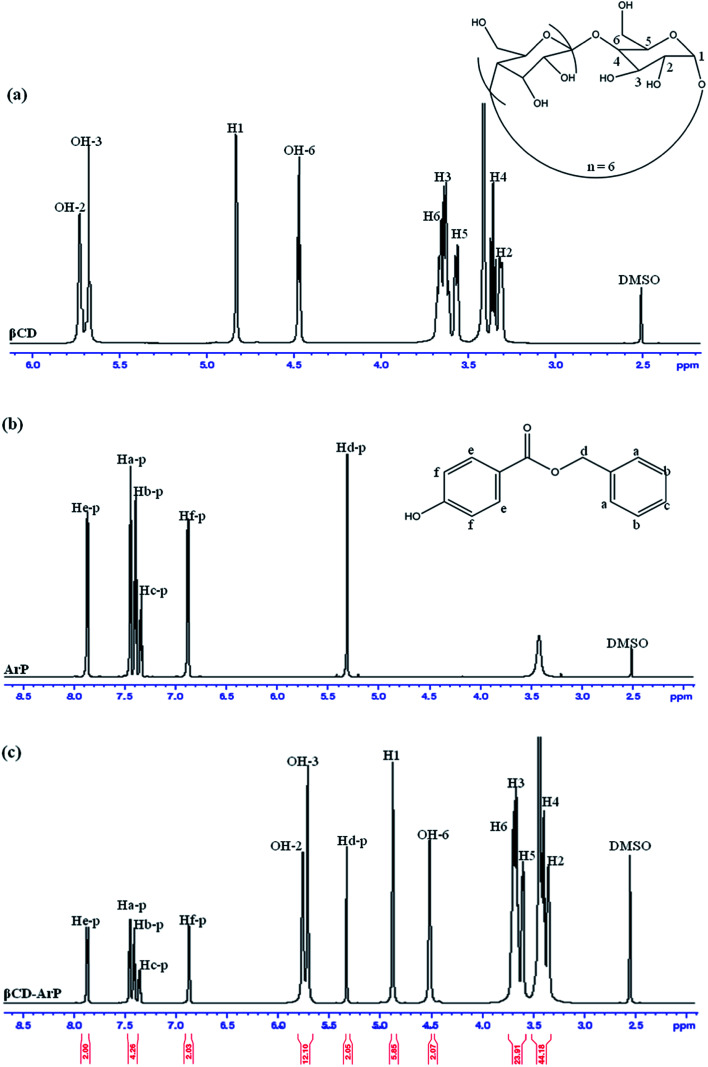
^1^H NMR spectra of (a) βCD, (b) ArP, and (c) βCD-ArP.

The formation of an inclusion complex can be confirmed by observing differences in chemical shifts (Δ*δ*) based on specific nuclei in the host molecule, because such changes in the microenvironment have been believed to occur in CD in inclusion complexes.^17^ The boldface values in Table 6 signify the changes in chemical shifts that took place in the reaction. The noted downfield shift for the protons on the inner cavity of βCD, *i.e.*, H3 and H5, corresponds to the encapsulation of the aromatic ring of ArP, which penetrated into the cavity of CD. Moreover, the H4 and H6 protons of βCD are located on the outer part of the cavity but also exhibited a downfield shift, except that they did not exhibit substantial changes upon the encapsulation of the aromatic ring of ArP. Furthermore, changes in the microenvironment of ArP protons led to upfield shifts for Ha-p, Hc-p, Hd-p, He-p, and Hf-p, whereas a downfield shift was observed for Hb-p among these protons. These protons belong to the aromatic rings that are present in the structure of ArP, as illustrated in Fig. 10b. Hence, the ^1^H signals of both βCD and ArP strongly suggest the formation of inclusion complex interactions, which can be clearly observed in the ^1^H NMR spectrum of βCD-ArP.

**Table 6:** Chemical shifts (*δ*) of βCD, ArP, and βCD-ArP

	βCD (*δ*)	ArP (*δ*)	βCD-ArP (*δ*)	Δ*δ*
H1	4.832		4.837	+0.005
H2	3.313		3.313	+0.000
H3	3.634		3.644	**+0.010**
H4	3.351		3.360	**+0.009**
H5	3.620		3.630	**+0.010**
H6	3.648		3.658	**+0.010**
Ha-p		7.440	7.437	**−0.003**
Hb-p		7.393	7.399	**+0.006**
Hc-p		7.347	7.345	**−0.002**
Hd-p		5.298	5.291	−0.007
He-p		7.863	7.856	**−0.007**
Hf-p		6.871	6.862	**−0.009**

### 3.8 Spectroscopic study

#### 3.8.1 Absorption spectrum of βCD–ArP complex

**Fig. 11:**
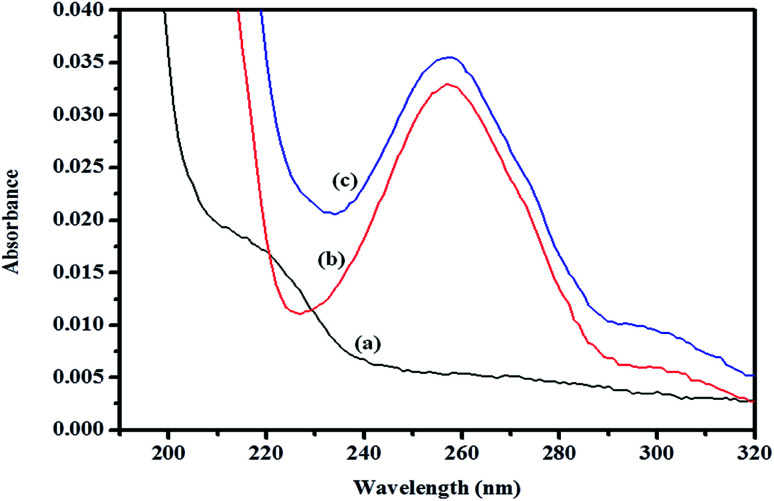
Absorption spectra of (a) βCD, (b) ArP, and (c) the βCD–ArP complex with [ArP]: 0.01 mM and [βCD]: 0.004 M at a pH of 7 and 25 °C.

**Fig. 12:**
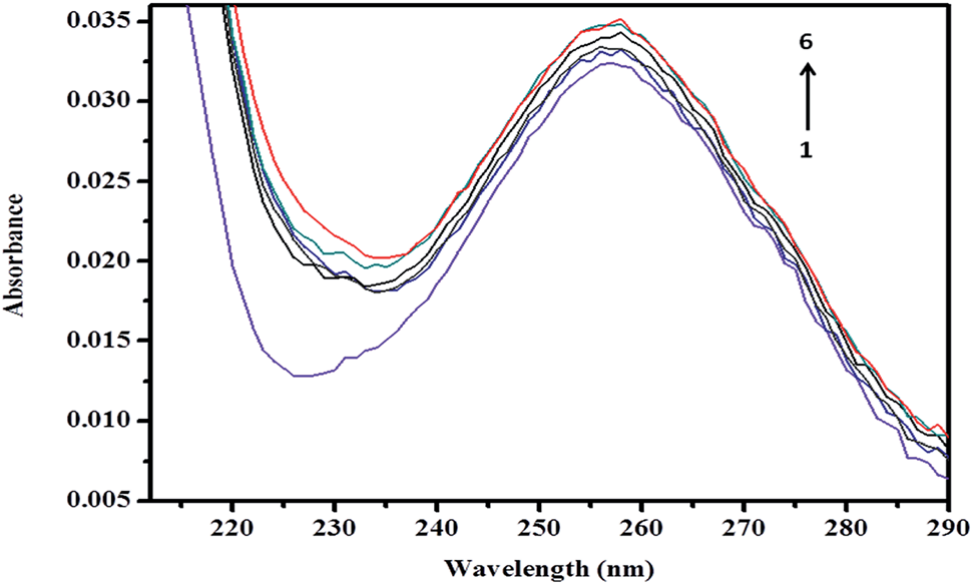
Absorption spectra of ArP (0.01 mM) with various concentrations of βCD at a pH of 7 and 25 °C. From line 1 to line 6: 0 M, 0.004 M, 0.005 M, 0.007 M, 0.009 M, and 0.01 M.

A spectroscopic study was further conducted to confirm the formation of the inclusion complex, as well as to determine the binding capacity between CD and ArP. The absorption spectra of the βCD–ArP complex, ArP, and βCD were recorded in accordance with the procedure described in Section 2.4.10. The results presented in Fig. 11 indicate that βCD exhibited no absorption in the range from 240 to 320 nm. In addition, the absorption spectrum of ArP seems rather similar to the spectrum of the βCD–ArP complex, except that the absorbance of the inclusion complex appeared to be higher than that of ArP at every wavelength. Furthermore, the influence of the βCD concentration on ArP was also studied, and the results are illustrated in Fig. 12. An increase in the βCD concentration led to a distinct variation in absorption intensity. Upon penetrating into the βCD cavity, the absorbance of the guest molecule was enhanced owing to the shielding of the excited species from non-radiative processes that occur in the bulk solution, together with an increase in the molar absorption coefficient of the inclusion complex.^17^

#### 3.8.2 Stoichiometry of the complex and the formation constant

**Fig. 13:**
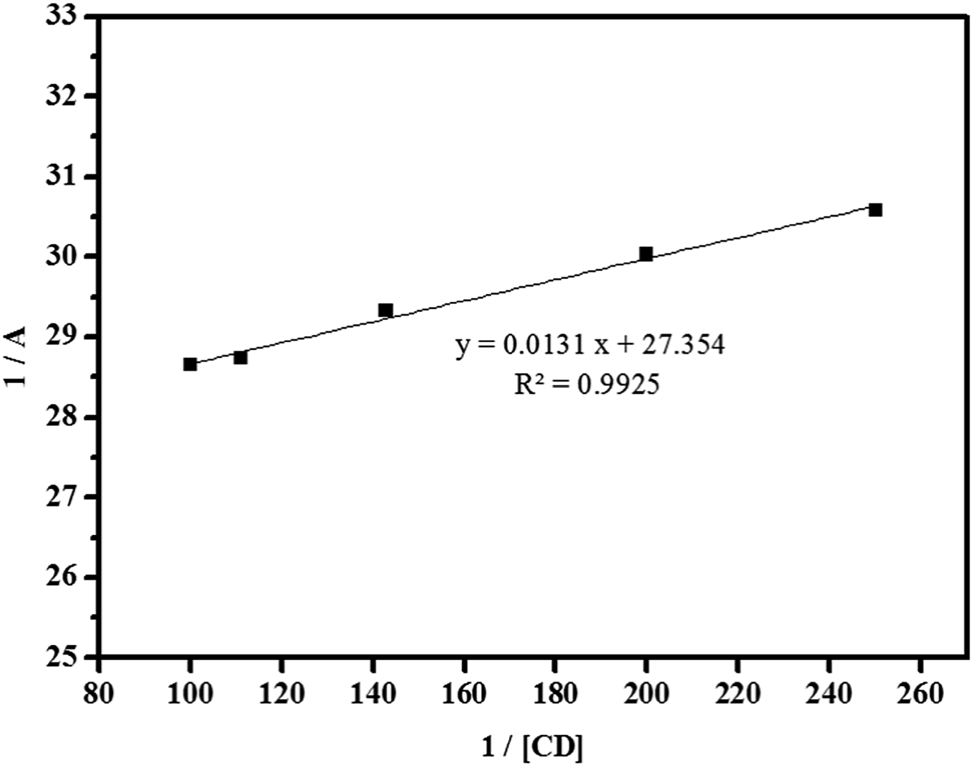
Reciprocal plot of 1/*A versus* 1/[CD].

The formation constant, which is denoted as *K*, was calculated by dividing the slope by the intercept of the straight line obtained in a reciprocal plot, whereas the stoichiometric ratio of βCD to ArP was determined using the Benesi–Hildebrand equation:^86^22
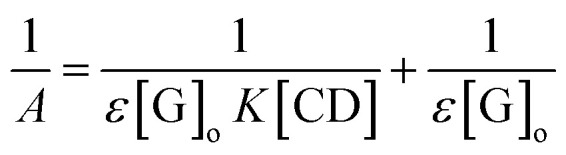
where *A* is the absorbance of the targeted analyte at each βCD concentration, [G]_o_ refers to the initial concentration of the targeted analyte, *K* denotes the apparent formation constant, [CD] represents the concentration of βCD, and *ε* is the molar absorptivity. Furthermore, the formation constant of the inclusion complex was determined by analysing the fluctuations observed in the absorption intensity with variations in the βCD concentration. The formation constant, which is denoted as *K*, was calculated by dividing the slope by the intercept of the straight line obtained in a reciprocal plot (Fig. 13). The graph, which was plotted with 1/*A* against 1/[CD], displays a good linear coefficient of determination, *R*^2^ = 0.9925, and thus signifies that the stoichiometric ratio of the complex that was formed is 1 : 1. In fact, the apparent formation constant is 2.09 × 10^3^ mol L^−1^.

## 4. Conclusion

In conclusion, IL-MNP-βCD-TDI was successfully tested as an adsorbent for the adsorption of selected paraben compounds, namely, PP, BP, and ArP. Furthermore, both kinetic and isotherm analyses demonstrated that the pseudo-second-order and Freundlich models provided better correlations for the adsorption of PP, BP, and ArP, with *R*^2^ values ranging between 0.980 and 0.999 for both models. In addition, both analyses were found to indicate that the equilibrium time was 80 min with an initial concentration of 80 mg L^−1^ at room temperature (298 K). Moreover, the negative value of Δ*H* demonstrated the exothermic nature of the adsorption process for all the studied parabens. In addition, the adsorbent was further analysed using real water samples. IL-MNP-βCD-TDI was also tested for the reusability of the sorbent, and it was found that the sorbent could be reused up to five times. Finally, an adsorption mechanism is proposed by considering the inclusion complex and π–π interaction between βCD and ArP, as proven *via* experimental analysis of ^1^H NMR spectra. Spectroscopic analysis also confirmed the formation of the inclusion complex by an enhancement in the absorbance of the guest molecule in UV-vis analysis. In brief, IL-MNP-βCD-TDI appears to be a promising material for the treatment of parabens in water.

## Conflicts of interest

All authors declare that there is no conflict of interest.

## Supplementary Material

RA-008-C8RA03408G-s001
