# Supplementary material for: A study on the removal of propyl, butyl, and benzyl parabens via newly synthesised ionic liquid loaded magnetically confined polymeric mesoporous adsorbent
Source: RSC Adv. 2018 Jul 18;8(45):25617–35. doi: 10.1039/c8ra03408g (PMC9082765; doi:10.1039/c8ra03408g)
Supplement: RA-008-C8RA03408G-s001 [file RA-008-C8RA03408G-s001.pdf]

Electronic Supplementary Material (ESI) for RSC Advances.
This journal is © The Royal Society of Chemistry 2018

List of supplementary information:

Fig. S1. IMAGE J analysis and SEM images of (a) MNP, (b) MNP-βCD-TDI, and (c) IL-MNP-βCD-TDI, as well as TEM images of (a') MNP, (b') MNP-βCD-TDI, and (c') IL-MNP-βCD-TDI.

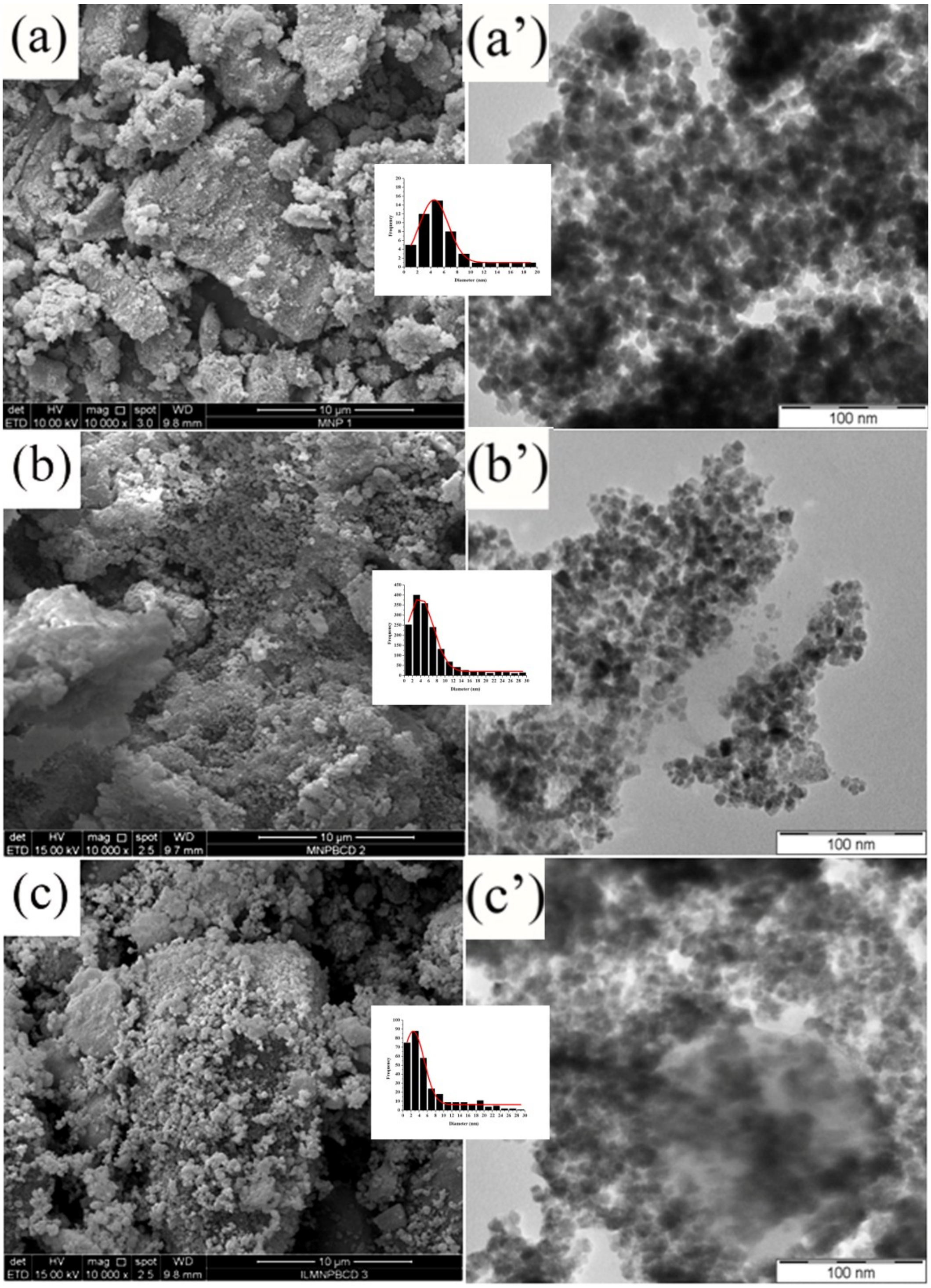

Fig. S1. IMAGE J analysis and SEM images of (a) MNP, (b) MNP-βCD-TDI, and (c) IL-MNP-βCD-TDI, as well as TEM images of (a') MNP, (b') MNP-βCD-TDI, and (c') IL-MNP-βCD-TDI.
